# CytoSpatio: Learning cell type spatial relationships using multirange, multitype point process models

**DOI:** 10.1371/journal.pcbi.1013409

**Published:** 2025-08-21

**Authors:** Haoran Chen, Yangyuan Zhang, Robert F. Murphy

**Affiliations:** Ray and Stephanie Lane Computational Biology Department, School of Computer Science, Carnegie Mellon University, Pittsburgh, Pennsylvania, United States of America; Radboud Universiteit, NETHERLANDS, KINGDOM OF THE

## Abstract

Recent advances in multiplexed fluorescence imaging have provided new opportunities for deciphering the complex spatial relationships among various cell types across diverse tissues. We introduce CytoSpatio, open-source software that constructs generative, multirange, and multitype point process models that capture interactions among multiple cell types at various distances simultaneously. On analyzing five cell types across five tissues, our software showed consistent spatial relationships within the same tissue type, with certain cell types like proliferating T cells consistently clustering across tissue types. It also revealed that the attraction-repulsion relationships between cell types like B cells and CD4-positive T cells vary with tissue type. Models for a published dataset demonstrated consistency with prior findings. CytoSpatio can also generate synthetic tissue patterns from learned models, a capability not provided by previous descriptive, motif-based approaches. This potentially allows spatially realistic simulations of how cell relationships affect tissue biochemistry.

## Author summary

A fundamental challenge in biology is to be able to accurately capture the processes by which different cell types are organized in different tissues. While this can be partially done by *descriptive* models that capture some spatial relationships between cell types, the ultimate goal is to be learn *generative* models that can create synthetic tissue images that are indistinguishable from real tissues. We have developed CytoSpatio, a generative model approach that is a significant step towards this goal, and have used it to create for the first time generative models of five human tissues that capture differences in the cell type spatial relationships among those tissues.

## Introduction

The functions of a tissue are often determined by the type and arrangement of its constituent cells. Distinct shapes, sizes, and molecular properties of cell types lead to specialized functions within a tissue [1–5]. However, spatial relationships among various cell types within diverse tissues are often more complex, and their impact on tissue functions is not fully understood.

Traditional imaging techniques, such as confocal microscopy, electron microscopy, and computed tomography (CT), have allowed scientists to investigate the spatial relationships between specific cell types within particular tissues [6–9]. However, these approaches typically required manual annotations of cell types. Therefore, they faced challenges of subjectivity in cell type annotations, limited scalability of conclusions across tissues, and most notably, the inability to capture complex spatial relationships due to the restriction on the number of identifiable cell types.

Recent advances in multiplexed imaging approaches for spatial transcriptomics and proteomics offer an unprecedented opportunity for researchers to explore the spatial relationships between a diverse range of cell types simultaneously [10–15]. By employing biomarkers targeting distinct RNA transcripts or proteins within cells in a multiplexed manner, various cell types can be concurrently visualized in tissue samples [16–18].

This advancement has motivated researchers to investigate spatial relationships among cell types with a variety of methods, mainly involving quantification and summarization of colocalization and correlation between cell types using analytic and statistical methods.

Behanova et al. [19] summarized and reviewed a variety of spatial statistics methods, tools, and software. The primary focus was on testing various hypotheses regarding whether cell types are randomly distributed, rather than attempting to construct models to capture complex spatial relationships.

A number of approaches for capturing these relationships have been described [15,20–25]. We discuss two illustrative approaches below.

Stoltzfus et al. [21] presented CytoMAP, a spatial analysis platform that quantified local cell composition and global tissue structure. This platform defines cell-centered local neighborhoods across the tissue, and groups similar neighborhoods together through clustering methods. It provides overall correlation and neighborhood composition between cell types for colorectal tumor and lymphoid tissues. While CytoMAP is a powerful tool for the spatial analysis of cell type relationships in tissue images, it is limited. Choice of the range for cell-centered local neighborhoods would be expected to significantly affect results, and only relationships within this range are captured.

Bhate et al. [24] hypothesized that tissues are composed hierarchically from smaller to larger components following certain assembly rules. To test this hypothesis, a hierarchical computational framework was devised to systematically identify the characteristic local compositions of cell types, known as cellular neighborhoods, map the local interactions and co-localization of these neighborhoods into distinct microenvironments, and delineate assembly rules that govern the formation of these microenvironments into tissue motifs. This hierarchical analysis produced proposed assembly rules for normal lymph node, spleen, and tonsil tissue, as well as colorectal cancer tissue. However, like CytoMAP, both the specific choices of the hierarchical design and the fixed parameters used to define the ranges of neighborhoods and microenvironments were not explored. The approach did not incorporate a probabilistic, generative framework to allow estimation of the likelihood of a tissue image being produced by a given model and/or the quantitative similarity between different tissues, or to allow generation of synthetic tissue images.

To address some of the limitations of existing methods that are primarily descriptive, we sought to employ *generative* statistical models to learn and represent the complex spatial relationships between different cell types in different tissues at different length scales.

Spatial point process models [26] are generative statistical models designed to learn the probability of individual objects (points) occurring at specific locations in space, including dependence of that probability on locations of other objects. The collection of points (including their locations within a defined region) are referred to as a “point pattern”, and models capturing how such point patterns are generated are referred to as “point process models.” These models have found widespread application in the analysis of spatial relationships across various domains, such as meteorology [27], ecology [28], criminology [29], and social sciences [30]. In cell biology, spatial point process models have been employed to elucidate the spatial relationships between punctate organelles and other cellular components, such as viruses, the nuclear membrane and microtubules [31–33]. They have also been used to investigate the assembly of viral ribonucleoprotein complexes [34] and to identify prognostic structural features in colon cancer tissues [35]. Although these point process models have been successful in revealing spatial dependencies and interaction patterns between objects in different contexts, they typically focus on one type of object at a time. In these models, the locations of other point types, if they exist, are treated as influential “factors” that may affect the spatial distribution of the target point type. Consequently, separate models must be trained for each object type. An alternate approach is the multitype point process model (or marked point process model) [36–40], which can simultaneously learn the spatial relationships between many types of objects (while typically limiting the factors that can be considered to inter-object distances). Such models have been used to identify gene expression patterns [41] and analyze cell-to-cell heterogeneity in tumor marker distributions [42].

In a multitype point process model, when assuming there are interactions between different types of points, a common challenge is to determine the maximum interaction distance over which two types of points can influence each other. Conventionally, a range parameter has been determined either by the distance of commonly observed interactions between two types of points [42] or by a distance distribution of nearest-neighbors between two types of points [33]. While these approaches offer a useful approximation, they are highly dependent on prior knowledge of likely interactions or the assumption that interactions are mostly limited to nearest neighbors. Choosing a different range for different datasets might also constrain the extent to which models trained on different tissues may be compared. To overcome this challenge, we introduce a novel approach to constructing multype, *multirange* models wherein different types of points can influence each other differently based on different ranges. This allows greater sensitivity in distinguishing different types of interactions, and is similar in principle to piece-wise linear, single type models [43].

**Fig 1:**
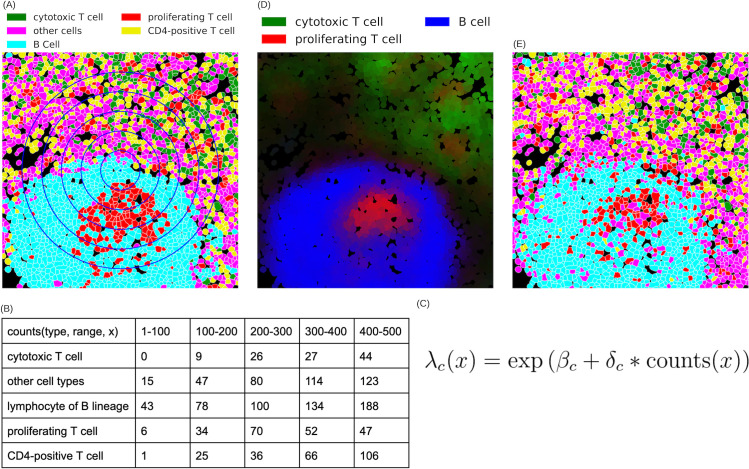
CytoSpatio process for learning spatial relationships between different cell types. **(A)** A region from a larger lymph node image is shown, with cell types shown in different colors and cell boundaries shown in white. The blue concentric circles denote five distance ranges of 100-500 pixels at 100-pixel intervals. **(B)** The training process involves counting the number of other cells of each type within varying distance ranges for each cell, as illustrated for the cell at the (small blue diamond) in panel A, a B cell. **(C)** A simplified version of the equation used for the fitting process in a point process model to learn the spatial relationships among cell types is shown. The probability λ of a particular cell type *c* at a given location, x, is given by a (global) base intensity (β) adjusted for the influence of (multiplied by) the local frequencies of all cell types. This adjustment is given by the dot product of a vector of interaction coefficients (δ) for this cell type with all cell types (including its own) and a vector (Counts(x)) reflecting the counts of each cell type. The interaction coefficient and counts can be for a single range (i.e., one of the columns in panel B) or can be concatenated across multiple ranges (i.e., linearizing the counts in panel B). **(D)** Predicted intensities (proportional to the probabilities of occurrence) are shown for three cell types for each cell in this region (derived from a model trained with the entire image). Brighter colors indicate a higher predicted intensity, with each color corresponding to a distinct cell type. **(E)** A synthetic image depicting predicted cell types generated for this region from the model is shown. The image was generated from the model using the positions of each cell in panel A but assigning each cell’s type based on the predicted probabilities across the cell types for that location (cell type colors are the same as in panel (A)).

We implement this approach in the open-source software package *CytoSpatio*. We demonstrate its superior performance over single-range models using images from five different tissues containing five distinct cell types. We show how the models can be used to compare cell type spatial relationships between images from the same tissue or between images of different tissues, and demonstrate both confirmation of previous findings and identification of new ones in previously studied tissue images. Additionally, we show how our approach can be used to evaluate heterogeneity in different tissue subregions. Perhaps most usefully, we construct interaction network graphs that directly exhibit and compare the spatial relationships among cell types. Lastly, we demonstrate generation of synthetic tissue images that capture cell-cell interaction relationships of training images. Fig 1 illustrates the processes involved in constructing models using our approach. We believe that CytoSpatio provides novel capabilities that can be complementary to existing tools for modeling cell-cell interactions.

## Results

For this study, we used multiplexed tissue images from the Human BioMolecular Atlas Program (HuBMAP) [44]. Images for five tissues were segmented into single cells and the cell type of each cell was assigned as described in the Materials and methods.

### Overview of modeling approach

The basic idea behind learning point process models is to fit a function that predicts the frequency of a particular event occurring at a location from information about the surroundings of that location. In basic multitype models, the event is the placement of a “point” of a given type, and the information is a tabulation of how many points of different types are nearby each point. In our case, points are cells, and we do not consider the size or shape of those cells. An example equation for such a model is shown in Fig 1c. The model itself consists of parameters (e.g., coefficients) that give the best fit given training examples. Further descriptions and equations for the different models used throughout are given in the Materials and methods.

### Assessing non-randomness of cell type distributions in different tissues

We began our analysis by exploring whether the cell type distribution in each tissue is random, which would imply a lack of meaningful spatial relationships among cell types. We posed a null hypothesis that the cell type distribution in a tissue image would be equivalent to a distribution with the same cell locations but randomized cell types. For each tissue, we randomized the cell types within all images 100 times, generating 100 sets of point patterns with shuffled cell types. These patterns served as a background distribution for our hypothesis testing. For each set, we trained a multitype Strauss Hardcore model (see Materials and methods) with the range that two cell types can affect each other (referred to as a Straus radius) set to 100 pixels and the range that two cells cannot come within each other (referred to as a Hardcore radius) set to 1 pixel (1 pixel equals 0.377 microns). The 100 pixel (38 microns) range was chosen to correspond to approximately 3–4 times a cell diameter. To measure agreement between a model and a set of point patterns, we used a metric that quantified the average disparity between each point pattern and the predicted intensity from the model (average deviance per cell, see Materials and methods). For each shuffled model, we measured average deviance per cell against a randomly selected shuffled point pattern set from the same tissue, and also against the unshuffled point pattern from the original image.

As shown in Table 1, we consistently observed that the average deviance per cell was lower when the models trained on a shuffled pattern set were tested against another shuffled point pattern set, as compared to when tested on the original point pattern set. We can estimate an empirical p value of 0.01 that the original pattern is non-random since we observed that all of the 100 shuffled patterns were closer to each other than any were to their original pattern (this was true for all five tissue types). Interestingly, we found that the cell type distributions in thymus, small intestine (SI), and large intestine (LI) were particularly non-random, resulting in significantly higher deviance when their shuffled models were tested against the original patterns.

**Table 1:** Comparison of average deviance per cell between shuffled point pattern sets and original point pattern sets. Lower average deviance per cell indicates a higher likelihood that a particular image could have been produced by a given model. The mean and standard deviation across the 100 shuffled patterns is shown on a log scale. The significantly higher deviances for the shuffled patterns and the original pattern demonstrate the non-random distribution of the cell types. How the extremely high deviances seen in some cases can be obtained is discussed in the Materials and methods.

	Shuffle-on-shuffle		Shuffle-on-original	
Tissue	mean	s.d.	mean	s.d.
Spleen	-0.69	0.0006	2.71	0.0896
Thymus	-0.65	0.0104	10.94	1.8544
Lymph nodes	-0.77	0.0050	1.89	0.1107
Small intestine	-1.24	0.0295	36.11	2.3915
Large intestine	-1.33	0.1243	38.58	2.9557

### Comparing multirange to single range of Strauss Hardcore

We next evaluated whether our multirange, multitype Strauss-Hardcore model (see Materials and methods) provides a more accurate fit for learning spatial relationships among cell types in our tissue images, compared to conventional Strauss Hardcore models with a single Strauss radius. For each tissue, we trained Strauss Hardcore models using various single radii (in pixels), as well as our multirange model that incorporates five distinct Strauss radii ranging from 100 to 500 at 100-pixel intervals (38–188 at 38-micron intervals).

An important component of constructing point process models is the creation of “dummy” points that have different types than the observed points so that the model can learn not only that observed points should have high probability for its observed type, but that observed points should in general have low probability for other types (see Materials and methods). In order to compare models for different radii, we evaluated each model’s goodness-of-fit using the average deviance per real cell, per dummy cell, and per both real and dummy cells.

**Fig 2:**
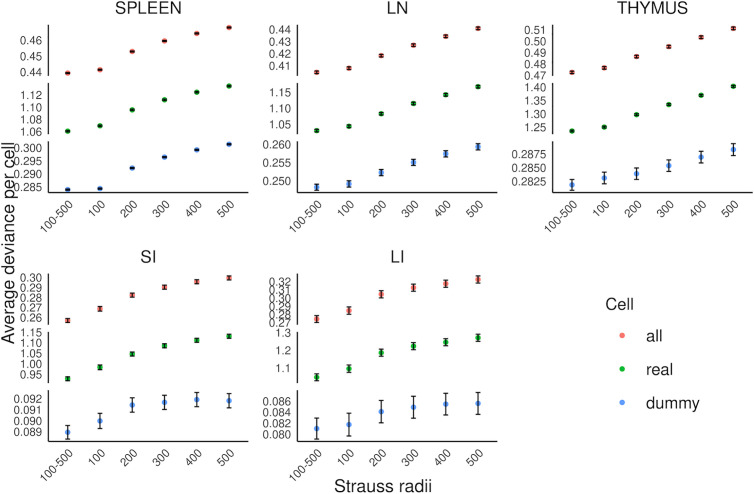
Performance comparison between multirange and single range multitype Strauss Hardcore models. The average deviance per cell for all cells, real cells, and dummy cells are shown (error bars show 95% confidence limits). The radii are in pixels, and correspond to 38 to 188 microns.

Fig 2 shows that, compared to the conventional Strauss Hardcore models with five single ranges, our multirange model consistently yielded the lowest average deviances for all five tissue types. Interestingly, we observed a gradual decline in the performance of the single radius model as the Strauss radius increased. This implies that the positioning of specific cell types is primarily influenced by their proximate neighboring cells, while cells at greater distances may introduce mixed spatial relationships that lower the prediction accuracy. Despite this, the spatial information derived from cells at larger distances remains beneficial for predicting cell types, contributing to the superior accuracy of the multi-range model across the five tissue types.

It is important to consider the relationships between the radius ranges used in constructing the models, the radii of the cell types being considered, and the size of the image pixels. For images with the same pixel size and similar cell radii, models can be directly compared (as we have here). As long as pixel size of the image (the width and height of each pixel in the sample plane; 0.377 microns for the images analyzed here) is reasonably smaller than the typical radii of the cell types, it does not significantly affect the estimation of cell-cell distances (when expressed in microns). Models for images of different pixel sizes can also be compared as long as the radius ranges (in pixels) are adjusted for each image so that they represent the same length in microns.

### Evaluating differences in cell type spatial relationships within and across tissues

We next asked, using two distinct approaches, how spatial relationships among cell types compare between different tissues. Both approaches used sets of models for each tissue that were derived from a leave-one-out cross-validation process (see Materials and methods).

**Fig 3:**
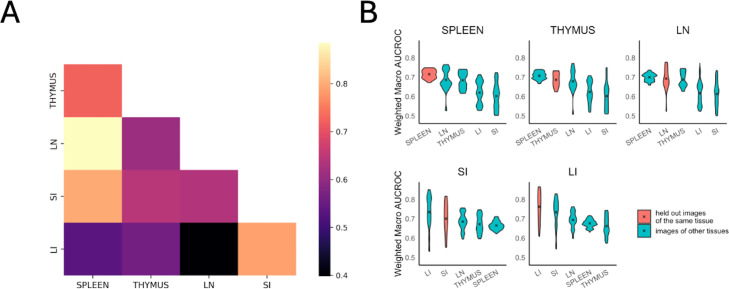
Comparison of cell type spatial relationships within and across different tissues. **(A)** The interaction coefficients between models are directly compared using Gaussian kernel similarity. Lighter color indicates greater similarity. **(B)** The predictive accuracy on held-out images of a given tissue as well as images from other tissues was measured using wmAUC. In each tissue panel, the violin plots are arranged in descending order of the mean from left to right, and the mean is indicated by an “x”.

The first approach involved calculating the Gaussian kernel similarity between the concatenated vectors of interaction coefficients for all radii (which encode the attraction or repulsion among cell types) of a pair of models. To provide an overall measure of similarity between tissues, we averaged similarity values between all pairs of models from two tissues (Fig 3A). We found that spleen, lymph node, and thymus tissues were more similar to each other than any of them were to either large or small intestine (which were quite similar to each other).

These distinct similarities and dissimilarities might reflect the organs’ primary biological systems and functions. The spleen, thymus, and lymph node are primarily part of the immune system, which could explain their high intra-tissue similarity. Conversely, the large and small intestines mainly serve the digestive system, but they also have immune functions. This dual role might contribute to the distinctive spatial relationships we observed between these two and the other three tissues.

For our second approach, the prediction accuracy of cell types was quantified using the weighted macro Area-Under-the-Curve (wmAUC, see Materials and methods). The results (Fig 3B) showed high (>0.7) values for all similarities between predicted and original cell types of the same tissue, especially considering the difficulty of predicting a single cell type only from the types of its neighbors. The highest value was not always that for a tissue with itself; this does not indicate poor performance of the model but rather reflects the similarity between particular tissues as already observed above. Spleen, thymus, and lymph nodes had a more consistent range of wmAUC values among images from the same tissue compared to those from the small and large intestines.

### Analyzing heterogeneity within tissue images

One assumption of point process models is that point patterns are homogeneous; in our case this means that spatial relationships among cell types remain consistent at different locations within the tissue. However, most tissues have distinct structural and functional units within them (such as stem cell niches). To evaluate whether such organization may be reflected in heterogeneity in cell spatial interaction models, we randomly segmented subregions (tiles) from the original images at two different sizes (5000x5000 and 2500x2500 pixels; 1888 microns or 942 microns in width). Tiles were required to contain at least 100 cells of all cell types and have at least one-fifth of the average number of cells per tile for that image. We ensured that the edges of each tile were at least 500 pixels away from the original image edges, since cells too close to the edge cannot have their interactions accurately counted.

For the same reason, we counted interactions for each cell within a tile with nearby cells outside the tiles. We preferred this approach to using an edge correction method since such methods are typically employed for individual images in which no information about the edge surroundings is available.

**Fig 4:**
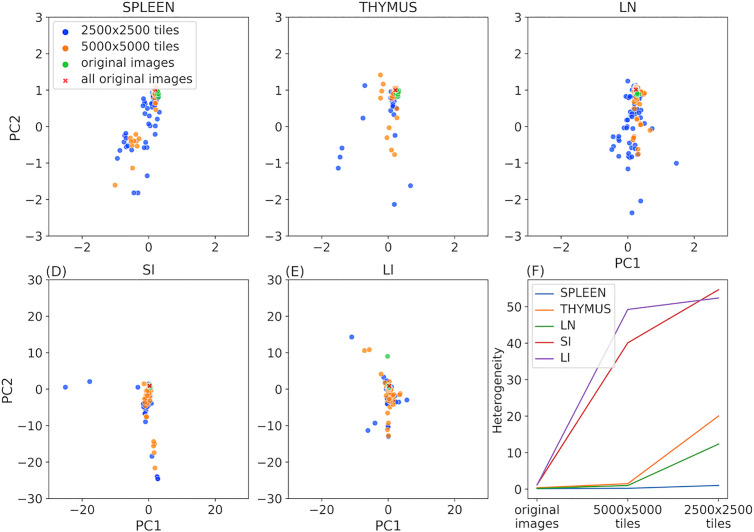
Evaluating tissue heterogeneity of cell type relationships. Panels A to E show the top 2 principal components of the interaction coefficients of various trained models for different tile sizes. Symbol colors are blue for 2500x2500 tiles, orange for 5000x5000 tiles, green for each original image, and red x’s for all original images combined. Panel F illustrates the change of heterogeneity with the tile size for the five tissues. Note that the PC1 and PC2 axis limits in panels A to C are ten times smaller than those in Panels D and E since there is much less variation in those tissues.

We trained and tested our model on each original image and tile, and for each tile size, we formed a matrix where each row represents a model for a given tile and each column corresponds to a interaction coefficient. Using principal component analysis, we extracted the two major modes of variation, enabling visualization of heterogeneity between individual tile models (Fig 4A–4E). We also transformed the interaction coefficients of the model trained on all original images of each tissue using the fitted PCA.

We also calculated the median of the Euclidean distances between the coefficients of models trained on tiles and coefficients of the model trained on all original images of that tissue. We used this value as a heterogeneity metric (Fig 4F).

Consistent with Fig 3B, spleen, thymus, and lymph nodes displayed lower heterogeneity across their original images compared to those of the large and small intestines. This homogeneity also persists for smaller subregions of those tissues (Fig 4A–4C) compared to intestine (Fig 4D and 4E). Fig 4F further quantifies this difference. It is of interest to note that within the three similar tissues, spleen exhibited a much smaller increase in heterogeneity for smaller subregions, suggesting largely homogeneous spatial relationships among cell types across various region sizes in this tissue.

### Visualizing cell type interaction networks

**Fig 5:**
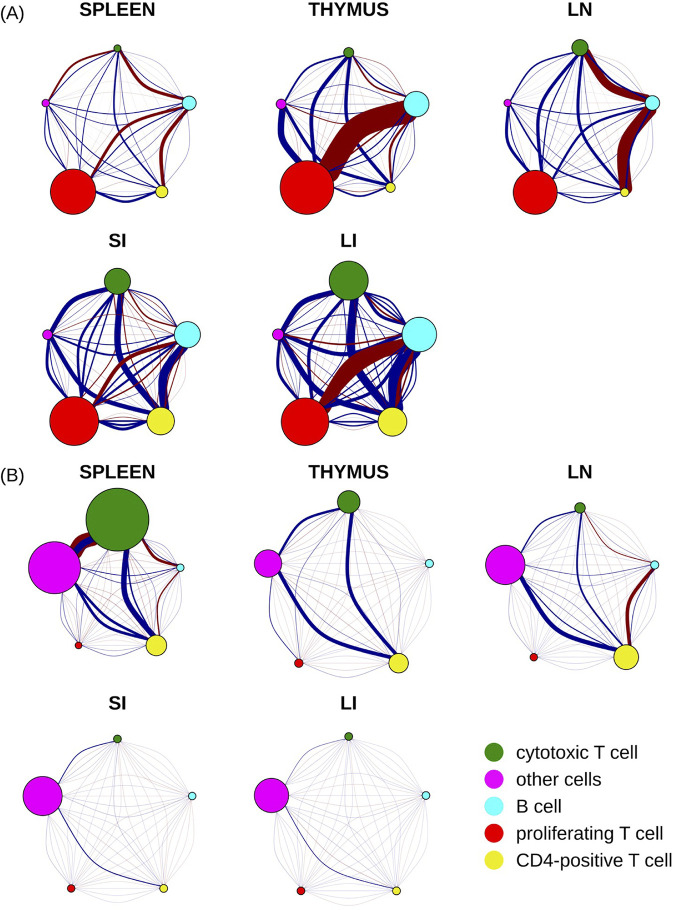
Cell type interaction graph for five cell types across five different tissues. The size of each node corresponds to the total strength of self-interaction across five distance ranges for that cell type (see S1 Fig for strength of self-interaction at each range). Each pair of nodes is interconnected by five arcs, each representing a different distance range. The range increases from left to right or from bottom to top, with the smallest and farthest ranges corresponding to the most curved arcs. The strength of the relationship between two cell types is depicted by the thickness of the arc, while the nature of their interaction is indicated by the color of the arc (blue as attraction and red as repulsion). **(A)** A direct, unfiltered illustration by raw interaction coefficients **(B)** Interaction coefficients adjusted by base intensities of corresponding cell types.

The primary goal of this study was to analyze the spatial relationships among cell types. To summarize our findings, we constructed interaction networks to visualize the interaction coefficients at various ranges in the multirange multitype Strauss Hardcore model (Fig 5).

We began by visualizing the interaction coefficients (δ) derived from models trained on all images for each tissue type (Fig 5A). These coefficients directly reflect the inherent probability that cell types are near each other, which for simplicity we can interpret as reflecting either “attraction” or “repulsion” between pairs of cell types. However, it’s crucial to emphasize that these inferred interactions aren’t based on isolated pairwise analyses for each pair of cell types. Instead, by integrating the interactions among all cell types in a single point process model, they represent interconnected behaviors between a pair of cell types factoring in influences from all other cell types concurrently.

Our analysis unveiled a variety of noteworthy interaction patterns among different cell types across several tissues. We detected a strong self-attraction among proliferating T cells throughout all the tissues studied (indicated by their larger node diameter). Conversely, cytotoxic T cells and CD4-positive cells demonstrated strong self-attraction in the small and large intestine tissues, but not in the other three tissues. B cells showed moderate self-attraction across all five tissues. As expected, the “other cell” type (cells that could not be annotated given the five markers common to all tissues), exhibited the weakest self-attraction. This is presumably due to the diversity of cell types within this category, with their respective influences offsetting each other.

As also expected, we found that the most intense interactions between two cell types generally occurred within the shortest distance ranges. However, there were a few notable exceptions. The interactions between cytotoxic T cells and B cells in small and large intestine, as well as between proliferating T cells and CD4-positive T cells in the large intestine, were moderate across a range of distances.

Our findings show high consistency between these interaction networks and the analysis presented in Fig 3B. When comparing the interaction networks for the small and large intestines, we discovered high similarity in both the direction of influence (attraction or repulsion) and the intensity of these interactions between cell types, with exception that B cells and proliferating T cells exhibited a notably stronger repulsion against each other within large intestine compared to their counterparts in small intestine. The spleen, thymus, and lymph node also demonstrated a high degree of similarity in terms of the direction of influence (attraction or repulsion) between cell types, but with variance in strength. For instance, thymus displayed stronger repulsion between proliferating T cells and B cells than the other two tissues. Lymph node had a stronger repulsion between B cells and both cytotoxic T and CD4-positive T cells, whereas the spleen demonstrated overall weaker interactions.

Our analysis also highlighted that in spleen, thymus, and lymph node tissues, B cells and CD4-positive T cells displayed a strong repulsive tendency at short distances (less than 40 microns), while they have a moderate attraction at larger distances. Interestingly, the interaction pattern between these two cell types reverses in large and small intestine tissues.

These conclusions are all made by examining the interaction coefficients (δ) directly, and thus assumes that the frequencies of the two types are approximately the same. However, it is worth noting that the extent to which a particular interaction is observed in tissue also depends on the base frequencies (β). Therefore, in contrast to “inherent” interaction coefficients presented in Fig 5A, we also calculated “apparent” interaction coefficients by multiplying them with the appropriate base intensities. These reflect the overall likelihood of observing a particular pair given their observed frequencies. As shown in Fig 5B, the likelihoods of all of the interactions of the “other cells” type were increased across all five tissues, due to the high frequency of that type. We found that the interaction likelihood of cytotoxic T cells in spleen also increased after adjustment. These cells exhibited the strongest repulsion with “other cells” at distances less than 100 pixels (<38 microns) and the strongest attraction at ranges between 100–200 pixels (38–76 microns). A universal attraction was observed across five tissues between cytotoxic T cells, CD4-positive T cells, and “other cells” with the attraction strength varyig. Furthermore, all cell types in small and large intestine, excluding “other cells,” displayed minimal likelihoods of either self-interaction or interactions among each other. This is consistent with the relatively low frequencies of these immune cell types in the small and large intestine tissues. It is important to keep in mind the distinction between “inherent” and “apparent” interactions: “inherent” interactions control for the confounding factor of cellular frequency to describe genuine interactions between cell types, whereas “apparent” interactions include cellular frequency to describe the ultimate patterning that arises in a tissue.

It is also important to note that Fig 5 shows all interaction likelihoods, even those that do not differ significantly from 0 (this can be determined since Cytospatio not only estimates interaction coefficients but also estimates the 95% confidence interval for each coefficient). CytoSpatio includes an option to omit insignificant interactions from the cellular interaction graph (such a graph corresponding to Fig 5A is shown in S2 Fig).

### Comparison to prior analysis on a larger number of cell types

**Fig 6:**
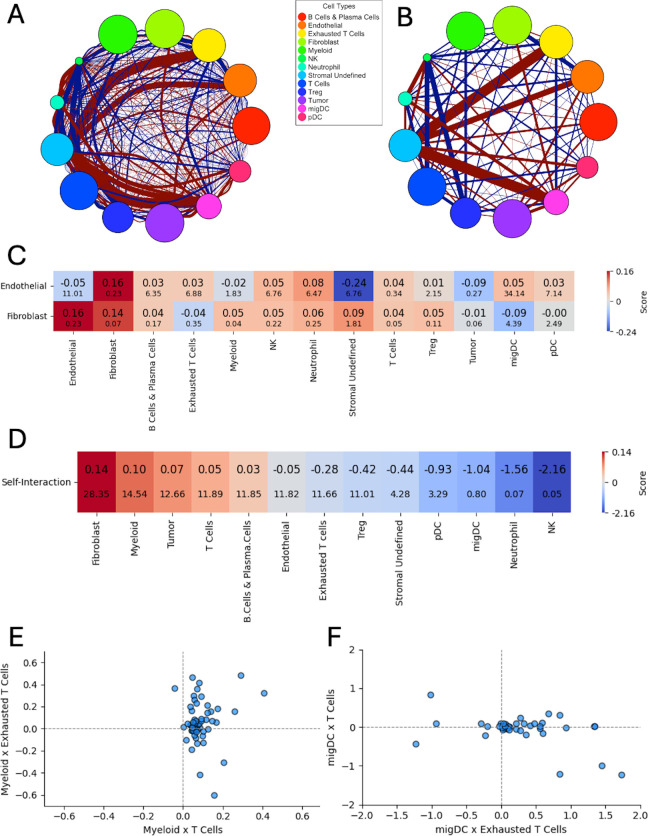
Results for IMC dataset. A, B) Interaction graphs similar to Fig 5A are shown for all five ranges (A) and for just the first range (B). **C)** Heatmap showing the pairwise spatial interaction scores between endothelial cells, fibroblasts, and other cell types. Each cell displays the 90% trimmed mean interaction score (top) and the Winsorized standard error (bottom) within the first distance range across 59 images. Among all combinations, the fibroblast–endothelial cell pair exhibits the strongest positive spatial association, with relatively low variability across samples. **D)** Similar heatmap for self-interactions for all cell types. Fibroblasts and myeloid cells exhibit the strongest self-interaction. **E)** Scatter plot comparing interaction scores between myeloid cells and PD-1 negative or positive T cells for 59 images. **F)** Scatter plot of interaction scores between migratory dendritic cells (migDCs) and PD-1 negative or positive T cells. Only images with scores the range (–2, 2) on both axes are shown to highlight the overall trend.

We sought next to compare results from our approach to those previously obtained on a human-annotated dataset. The availability of multichannel datasets with human-annotated cell types is quite limited. We therefore chose to apply CytoSpatio to a dataset of Imaging Mass Cytometry (IMC) data from tissue sections of 12 human breast tumors [45] that has previously been analyzed with HistoCAT [20]. The dataset includes cell type annotations of 19 cell types at three hierarchical levels. As described in Materials and methods, we consolidated these down to 13 that were present at sufficient levels to support our more extensive statistical modeling. Given the significant variation between the images (expected since they are from different tissue samples), we chose to construct models for each image. Fig 6A presents the overall interaction graph derived from the median coefficients of all regions, where (as in Fig 5) node sizes represent self-interaction strength and edges encode interaction direction and magnitude across five spatial bins. A wide range of both positive and negative interactions can be seen. (In view of the complexity, just the first range is shown in Fig 6B and the individual interaction coefficients for each range are shown in S3 Fig). We observed strong self-interactions among Tumor, Fibroblast, and Myeloid cells. Notably, migDCs (migratory dendritic cells) exhibited strong repulsion from Stromal Undefined cells, a previously unreported spatial exclusion pattern.

To examine whether CytoSpatio recapitulated key spatial patterns reported in the original histoCAT analysis, we focused on comparing interactions at the smallest spatial range (37.7 microns), which most closely mirrors the neighborhood-based analysis used by histoCAT. We specifically evaluated four patterns reported in Fig 6A in Tietscher et al [45].

Pattern 1 was a strong positive interaction between fibroblasts and endothelial cells. To assess this, we computed the trimmed mean (with 5% trimming on both tails) and Winsorized standard deviation of interaction scores across all 59 images (Fig 6C). The fibroblast–endothelial pair displayed one of the highest positive interaction scores with low variability, in line with the original findings. Pattern 2 was that myeloid cells display high self-interaction. We assessed self-interaction across all cell types (Fig 6C) and found that Fibroblasts had the highest self-interaction score, followed closely by Myeloid cells—both consistent with histoCAT-reported clustering behavior.

Patterns 6 and 7 (respectively) were that myeloid cells preferentially interact with PD-1 negative T cells over PD-1 positive T cells, and that migDCs show the opposite preference. To evaluate these, we created scatter plots of interaction scores for each image for myeloid × PD-1 positive T cells versus Myeloid × PD-1 negative T cells (Fig 6E), and migDC × PD-1 negative T cells versus migDC × PD-1 positive T cells (Fig 6F). These plots illustrate the significant variation among the different tissue images. Nonetheless, most points in Fig 6E lie to the right of the vertical axis, indicating that Myeloid cells exhibit stronger interactions with T cells in general. The lack of a clear vertical trend suggests no preferential interaction with PD-1 positive T cells, which supports Pattern 6. The p value of 0.031 was obtained for the hypothesis that the interaction is not stronger with PD-1 negative T cells than PD-1 positive T cells using the Wilcoxon test. Similarly, the majority of points in Fig 6F fall to the right of the vertical axis, indicating that migDCs preferentially associate with PD-1 positive T cells. The absence of a vertical trend further supports Pattern 7. A p value of 0.008 was obtained for this result.

Notably, our analysis also uncovered a previously unreported spatial pattern that was not identified using histoCAT. As shown in S3 Fig, certain immune cell types—most prominently NK cells, but also Neutrophils and pDCs—exhibited consistently negative self-interaction scores across all spatial distance ranges. Upon manual inspection of the tissue images, we observed that NK cells were generally sparsely distributed, often appearing as isolated cells or forming small clusters of two or three cells. This spatial pattern is structured and non-random, yet does not meet the definition of clustering. In contrast, histoCAT analysis assigned these cells relatively high self-interaction scores, likely failing to distinguish between small scattered doublets and true dense clusters. We hypothesize that this discrepancy arises because histoCAT relies primarily on nearest-neighbor statistics, which are sensitive to the presence of adjacent cells but not the broader spatial context. In scenarios where scattered doublets or triplets are distributed across space, nearest-neighbor methods may incorrectly interpret them as evidence of clustering. CytoSpatio, by contrast, explicitly models spatial associations across multiple distance ranges. As such, our framework can more accurately capture nuanced spatial arrangements, such as the dispersed but locally paired distribution of NK cells.

### Simulating artificial tissue images from generative models

Perhaps the most valuable property of a generative model lies in its ability to create new samples based on its learned probability density functions. We therefore asked whether our models could generate artificial tissue images that maintain their learned spatial relationships among cell types.

To do this, we generated images from our models using two methods described in Materials and methods. Both start with cell positions chosen from a Poisson distribution with the same total cell density as an original image. The difference lies in how cells are assigned cell types. We focus here initially on method 1, which involved making initial cell type assignments at random and then iteratively selecting a cell and reassigning its type according to the cell type counts for that location and the likelihoods derived from the model (the model in this case was for one original image of each tissue). This process was continued until the number of sampled cells reached a specified percentage of the total cell count in the image. We conducted separate trials with different random seeds, and for each trial sampled cells from 0 to 400 percent of total cell counts in intervals of 50 percent. We measured the wmAUC of the original model with respect to the synthetic images, which reflected how well the arrangement of the assigned cell types agreed with the model. We expected that the reassignment process would result in increased wmAUC as it converged as cell type assignments in agreement with the model.

As shown in S4 Fig, the wmAUC nearly monotonically increased with the resampling percentage. This observation suggests that our model is capable of generating synthetic images with cell type spatial relationships similar to those in the original images, although the wmAUC values are a bit lower than those obtained for predicting individual cell types in original images. Even higher accuracy synthetic images could presumably be generated by using even more resampling for different random seeds and choosing the one whose coefficients are most similar to those of the model.

We also generated synthetic images using method 2; rather than making initial assignments to all cells and then refining them, it iteratively assigns types to randomly chosen cells according to the model predictions. We generated baseline images as well using two approaches. As a rough independent means of comparing the synthetic images generated by these approaches to real images, we measured the frequencies of cell types within 100 pixel radii. The results (S5 Fig) indicate that a more gradual generation approach (method 2) gave better agreement with real images that method 1, and that generating simply according to cell type frequencies provided similar performance on this rough measure. This is likely because the rough measure does not take into account the multiple radius properties that our models capture.

**Fig 7:**
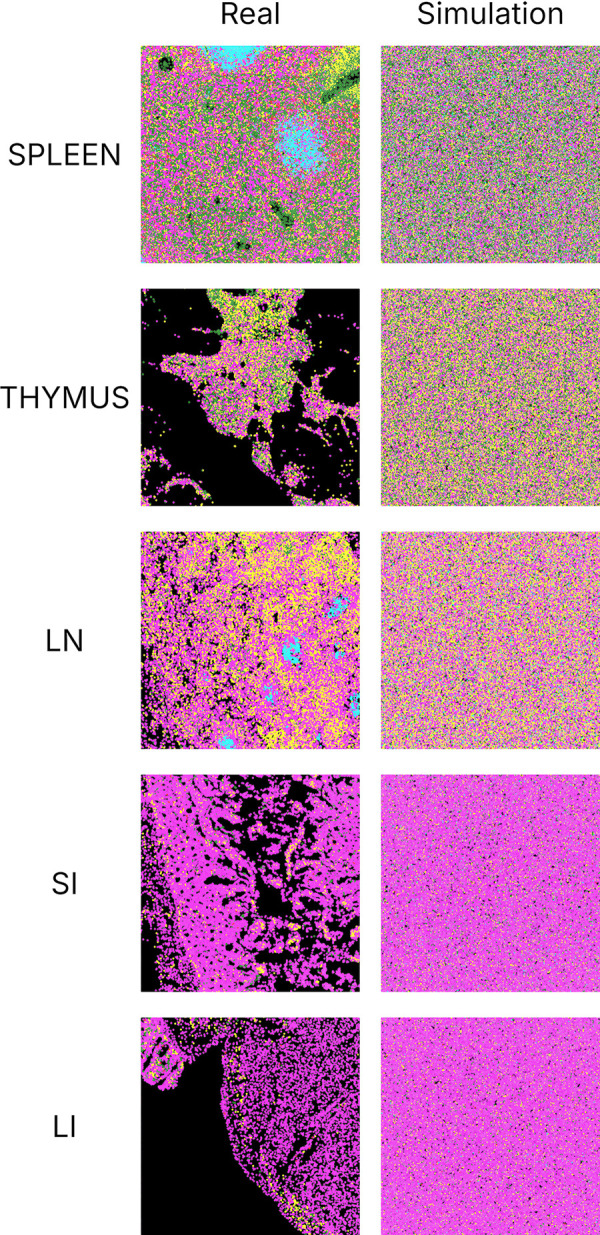
Real and synthetic tissue images across five tissue types. Synthetic images were generated using method 2 (see Materials and methods). Each color represents a unique cell type, consistent with representations in other figures.

Fig 7 shows how our models can be used to illustrate the differences in cell type arrangement that would result for different tissues if cell locations and sizes were kept constant. Synthetic images generated by method 2 are compared with a corresponding real image region for each tissue. Since the generation process does not account for the distribution of cell positions within a tissue (e.g., regions with no cells such as lumens and blood vessels), the real images are clearly distinguishable from the synthetic ones. However, the synthetic images do reflect the trends captured by the adjusted interaction coefficients in Fig 5B for all spatial relationships between cell types, including self-interactions. In particular, the tendency of cytotoxic T cells to be near each other is preserved in all tissues even as the frequency of those cells changes. Cytotoxic T cells and CD4-positive T cells are consistently found near each other across three immune tissues spleen, thymus, and lymph node. This proximity is consistent with their high attraction as represented in Fig 5B. In lymph node synthetic tissue, B cells and CD4-positive T cells exhibit repulsion at short distances whereas attractive to each other at longer distance, aligning with the observations in Fig 5B. While B cells generally appear to be repulsive to both CD4-positive T cells and cytotoxic T cells at short distances in spleen tissue, exceptions can be found Fig 7. This may be attributed to the high intensity of both cytotoxic T cells and CD4-positive T cells in spleen. In both small and large intestine tissues, fewer B cells and T cell types are observed, which is consistent with the low “apparent” interaction strength between these cell types depicted in Fig 5B after adjustment for cell intensity. Nevertheless, we were able to discern the inherent interactions between these cell types in these two tissues, as illustrated in Fig 5A.

## Discussion

Spatial relationships among cell types are critical determinants of tissue functions. In this study, we present CytoSpatio – open-source software that constructs innovative generative multitype, multirange point process models to learn spatial relationships between cell types. Our model is built upon a baseline multitype Strauss Hardcore model, incorporating multiple ranges of Strauss radii in a piece-wise manner that captures diverse properties of both signs and strengths of interactions among cell types at varying distances. We demonstrated that our model successfully captures differences in images from different tissues (Fig 3). Additionally, we describe a quantitative approach for assessing spatial heterogeneity within a tissue, and found differences among the five tissues (Fig 4). To visualize the spatial relationships of cell type, we constructed interaction networks and discussed the similarities and differences across 5 tissues (Fig 5). We also applied our approach to a previously analyzed dataset and both confirmed results (Fig 6) and obtained a novel finding regarding NK cell distribution (S3 Fig). Furthermore, we showcased the capability of our model to generate synthetic tissue images that reflect the spatial relationships among cell types in the original tissue images (Fig 7).

Our approach has the advantage that it can be used even on single images. The IMC images we have modeled have around 50,000 cells with the smallest subtypes (NK cells and micDC cells) having an average of 40 cells per image. While this may be useful when considering using CytoSpatio, successful modeling for specific images may depend not just on the number of rare cells but on their spatial distribution.

We demonstrated that our multirange, multitype model provides enhanced capabilities for capturing complex spatial relationships among cell types, achieving a balanced trade-off between computational complexity and the ability to learn spatial relationships. The multirange capability confers advantages beyond avoiding the need to choose a particular interaction radius. In principle, one could run other tools (such as CytoMAP) one range at a time for a set of ranges. However, these separate models would not capture relationships *between* ranges.

Importantly, our multitype, multirange models can capture relationships beyond pairwise cell type interactions. Since the contribution of a particular range to the overall predicted probability of a given cell type at a given location is a product of the interaction terms for cell types occurring in that range, we can capture “multipartner” relationships such as cell type A attracting cell type B in the presence of cell type C but repelling it in the absence of cell type C. Similarly, changes in the density of one cell type between two or more ranges can cause nonlinear effects on the probability of another. Explanations of these multipartner and nonlinear effects are presented in the section “Properties of the model” in Materials and methods, and examples of multipartner effects are listed in S4 Table.

In our studies, we allowed a maximum range of 500 pixels, or approximately 188 microns, as the distance within which two cells could affect each other. As shown in S3 Table and S6 Fig, an example examination of the effect of decreasing the interval showed little effect on the conclusions. As the first interval went from 50 to 100–200, the β values (which set the expected intensity for each type) stayed very constant, as expected since they reflect the overall frequencies. The interaction coefficients for the first interval changed somewhat but remained highly correlated, as expected given that most of the interactions observed in our models above were in the first distance range. The interaction graphs in S6 Fig show the same interactions as being attractive or repulsive. However, other tissues or cell types may show different range properties, and CytoSpatio easily allows user specification of the maximum range and interval to explore this. Furthermore, there is room for refining our model’s interaction function, which currently exhibits a sudden shift of influence every 100 pixels, or approximately 38 microns, due to the piece-wise step function (see Materials and methods). The intervals of our current interaction function could benefit from optimization, and interaction functions with smooth transitions such as Softcore, Fiksel [46], Diggle-Gratton [47], Diggle-Gates-Stibbard [48] might also be worthwhile to explore. In addition, models capturing higher order interactions such as area-interaction [49] and Geyer saturated model [50] where the interaction functions are determined by the relationships of three or more points may be valuable. Currently, the lack of availability of software supporting the multitype versions of the interaction functions limits their use, but future implementations could enhance the representation of interactions among cell types in different scenarios.

A related approach to generating simulated tissue images was published [42] while this work was being written up for submission. Unlike our approach, it requires user specification of cell type proportions and a pairwise neighbor probability matrix, rather than learning these from images.

Recently, multiplexed tissue imaging technologies have been extended to high-resolution, three-dimensional images [51]. The addition of a third dimension significantly increases the complexity of spatial relationships among cell types and the challenges associated with modeling these relationships. Consequently, there is an urgent need for 3D multitype point process models, since building models on 2D slices or 2D-projections may not capture relationships accurately. We are currently extending our pipeline to model 3D cell type spatial relationships, aiming to deepen our understanding of their impact on tissue function in a 3D context.

Our study successfully depicted the spatial relationships among five cell types in five distinct tissues, with a majority being immune cell types. Rather than making the traditional assumption that these cell types (e.g., B cell, T cell and their subtypes) are generally located near one another for close collaboration [52,53], we have quantitatively examined their attraction and repulsion tendencies across varying distances. For example, we found a strong preference against B cells and proliferating T cells being closer to each other than ~38 microns in spleen, thymus, small and large intestine tissues but the opposite tendency at larger distances. Our approach can not only challenge existing qualitative perspectives on spatial relationships among immune cell types but can also potentially provide valuable quantitative insights into how cell types assemble to form tissues.

It is important to note that the synthetic images generated by CytoSpatio do not capture all aspects of cell relationships in tissue images. We are in the process of upgrading CytoSpatio’s simulations to better capture the arrangement of cell positions and to include cell shape. For the latter, we require a generative model capable of learning and simulating diverse cell shapes. In this regard, a robust version of spherical harmonic transform parameterization has been demonstrated as the most effective and accurate method for generating cell shapes [54]. Steps such as these will hopefully allow construction of more comprehensive and detailed representations of tissue images.

## Materials and methods

### CODEX tissue images and cellular data

We used 110 images from the Human BioMolecular Atlas Program (HuBMAP) consortium [44] that had been acquired using the CO-Detection by indEXing (CODEX) [15] method. A summary of these images is provided in S1 Table. They were produced by two Tissue Mapping Centers (TMCs): Stanford TMC produced images of the large and small intestine with 47 fluorescence channels (markers), and the University of Florida TMC produced images of the lymph node, thymus, and spleen with 11 fluorescence channels. Image sizes vary, ranging from approximately 5,000–15,000 pixels, with each pixel corresponding to a tissue region of 0.37745 x 0.37745 microns. The images share five common channels (CD11c, CD21, CD4, CD8, Ki67) across both TMCs. We downloaded files detailing the total intensities of the cell boundary, cytoplasm, nuclear boundary, and nucleus of each channel and the coordinates of cell centers from the HuBMAP portal (https://portal.hubmapconsortium.org/). These files were generated using SPRM (https://github.com/hubmapconsortium/sprm), based on cell segmentations created by Cytokit [55].

### IMC images with assigned cell types

We also analyzed a dataset of Imaging Mass Cytometry (IMC) images derived from tissue sections of 12 human breast tumors created by Tietscher et al [45]. It consists of 77 IMC images with a pixel size of 1 micron. Cells in these images were classified into three hierarchical levels: *cell_class* (2 categories), *cell_type* (11 categories), and *cell_subtype* (19 categories). A file containing cell positions and cell type labels for all images (filename “Protein_panel_singlecell_metadata.csv”) was downloaded from DOI 10.5281/zenodo.4911135. We merged the four tumor subtypes into one *tumor* type, PD-1 high CD4 and CD8 T cells into one *PD-1 positive T cell* type, and PD-1 low CD4 and CD8 T cells into one “normal” *PD-1 negative *T cell** type. We excluded 16 of the 77 images which did not have all 13 types present, and 2 images failed in model building due to training divergence, resulting in 59 images used for subsequent analysis.

### Assigning cell types for CODEX images

Different cell types typically express varying levels of specific cell marker proteins. For instance, proliferating T cells demonstrate high Ki67 levels and low levels of other markers, whereas cytotoxic T cells exhibit high CD8 levels. We defined cell types based only on the five common channels to ensure comparability across tissue types. This decision allows direct comparison of spatial relationships among cell types across various tissues in subsequent analyses.

To compensate for potential differences in channel intensities across tissues, such as those that might arise during image acquisition due to experimental variables like inconsistencies in staining procedures or tissue preparation, we initially z-scored total pixel intensities per cell for each channel within each tissue.

For cell type assignment, we first performed KMeans clustering on the total pixel intensities per cell over the z-scored five common channels across all cells and images from the five tissues. Next, we calculated an overall similarity statistic T based on Gaussian Kernel similarity for intensity compositions of cells between 1) each pair of clusters from KMeans and 2) each cluster from KMeans and each annotated cell type from a lymph node image annotated by Cellar [17] (S7 Fig). Using these results as features, we conducted another round of KMeans as meta-clustering to assign the clusters to the five cell types annotated by Cellar.


$$T=\frac{1}{m^{2}}\sum_{i=1}^{m}\sum_{j=1}^{m}K\left(X_{i},X_{j}\right)-\frac{2}{mn}\sum_{i=1}^{m}\sum_{j=1}^{n}K\left(X_{i},Y_{j}\right)+\frac{1}{n^{2}}\sum_{i=1}^{n}\sum_{j=1}^{n}K\left(Y_{i},Y_{j}\right)$$ (1)

$$K(x\text{,}y)=\text{exp}\left(-\frac{{\left||x-y|\right|}^{\text{2}}}{\text{2}\sigma^{\text{2}}}\right)$$

where T is the statistic measuring overall similarity between two cell types, lower T indicates higher similarity. *m* and *n* are the number of cells in two cell types, respectively. $X_{i}$ and $Y_{j}$ indicate the cell intensity composition of *i*^*th*^cell in cell type *X* and *j*^*th*^ cell in cell type *Y*. *K* is the Gaussian kernel similarity and σ is the bandwidth of the kernel (we used $\text{2}\sigma^{\text{2}}=\text{0}.\text{08}$; this value was also used for other Gaussian kernel similarity measurements).

To determine the optimal number of clusters in the initial KMeans, we incrementally increased the number of clusters while monitoring the number of cells in each assigned cell type. We then selected the number of clusters that yielded the highest match between assigned cell types and their corresponding cell types from Cellar (S8 Fig). We note that this approach enables the extrapolation of cell type determination from lymph nodes to other tissues, and it allows for finer distinctions within each cell type (i.e., the identification of potential cell subtypes).

For simplicity, all cells assigned to the type “lymphocytes of B lineage” are referred to throughout as simply “B cells.”

### Point pattern and point process model

For each image across 5 tissues, we formed a point pattern $p\;=\;\left\{\left(x_{1},c_{1}\right),\ldots ,\left(x_{i},c_{i}\right),\ldots ,\left(x_{n},c_{n}\right)\right\}$, where $x_{i}\;$ is a vector of 2-dimensional coordinates (i.e., cell center) for cell *i*, $c_{i}$ is the cell type of cell *i* and *n* is the total number of cells in the image. The coordinates were defined separately in each image. The point patterns belonging to each tissue were considered as random realizations (instances) from a point process model. Our task was to define this point process model.

We assumed cells influence each other by both attraction and repulsion. Therefore, we chose to use the multitype Strauss Hardcore model [26], a kind of multitype Gibbs model, as our baseline model since it satisfies this assumption and can model all cell types at once. The model consists of an expression that allows estimation of the probability density *f*(***p***) of a given point pattern given a set of model parameters (that is, the probability that a particular point pattern would have been observed given those parameters)


$$f\left(p\right)=\alpha \;\;\prod {\mathrm{\backslash nolimits}}_{i=1}^{n}\beta_{c_{i}}\left(x_{i}\right)\prod {\mathrm{\backslash nolimits}}_{i<j}^{n}\gamma_{c_{i},c_{j}}\left(d\left(x_{i},x_{j}\right)\right)$$ (2)

where *f* is the probability density of point pattern ***p***, α is a normalizing constant, $\beta_{c_{i}}$ is the intensity of cell type $c_{i}$ of point $x_{i}$, *n* is the total number of cells in the pattern, $\gamma_{c_{i},c_{j}}$ is the interaction function between cell type $c_{i}$ and $c_{j}$, $d\left(x_{i},x_{j}\right)$ is the Euclidean distance between cell $x_{i}$ and $x_{j}$. From this we can also write an expression for the conditional intensity (probability) of finding a cell of cell type $c_{i}$ at location $x_{i}$ given the point pattern ***p***


$$\lambda \left(\left(x_{i},c_{i}\right)p\right)=\beta_{c_{j}}\left(x_{j}\right)\prod_{j=1,\left(x_{i},c_{i}\right)\neq \left(x_{j},c_{j}\right)}^{n}\gamma_{c_{i},c_{j}}\left(d\left(x_{i},x_{j}\right)\right)$$ (3)

which ignores any contribution from the actual type of that cell.

The interaction function encodes the spatial relationships between two cell types. In multitype Strauss Hardcore model, the interaction function is


$$\gamma_{c_{i},c_{j}}\left(d\left(x_{i},x_{j}\right)\right)=\left\{ \begin{array}{c} 0\;d<r_{h}\\ \delta_{s}\;{\;r}_{h}\leq d\leq r_{s}\\ 1\;d>r_{s} \end{array}\right.\;$$ (4)

where $r_{h}$ is the hardcore radius that specifies the minimum distance that two cells can be from each other, $r_{s}$ is the Strauss radius which represents the maximum distance over which cells can affect each other, and $\delta_{s}$ is the interaction coefficient that captures whether two cells may have attraction $\left(\delta_{\text{s}}\;>\;1\right)$ or repulsion ${(\delta }_{\text{s}}\;<\;1)$ between each other.

One limitation of the conventional Strauss Hardcore model is that the influence between cells is uniformly across a certain single range (Strauss radius $r_{s}$), whereas for given spatial relationships between two cell types it may actually vary with distance. To address that, we proposed a multirange multitype model with an upgraded piece-wise interaction function [56]:


$$\gamma_{c_{i},c_{j}}\left(d\left(x_{i},x_{j}\right)\right)=\left\{ \begin{array}{c} 0\;d<r_{h}\\ \delta_{s_{1}}\;{\;r}_{h}\leq d<r_{s_{1}}\\ \delta_{s_{2}}\;{\;r}_{s_{1}}\leq d<r_{s_{2}}\\ \ldots \\ \delta_{s_{m}}\;{\;r}_{s_{m-1}}\leq d\leq r_{s_{m}}\\ \;1\;d>r_{s_{m}} \end{array}\right.\;$$ (5)

where different interaction coefficients $\delta_{s_{\text{1}}}\ldots \delta_{s_{m}}$ are assigned to each distance interval. For each pair of cell types, we have $\delta_{c_{i},c_{j}}=\left(\delta_{s_{\text{1}}}\ldots \delta_{s_{m}}\right)$, which is the same for all interactions between cell type $c_{i}$ and $c_{j}$, where $c_{i},c_{j}\in C$, C is the set of all cell types.

### Properties of the model

As shown in equation 3, the probability of a given cell type at a given location is proportional to the product of the pairwise interaction coefficients of that cell type and the cell types of all other cells. This gives rise to two useful properties.

First, even though the coefficients are pairwise, the model can capture interactions between more than two cell types. To illustrate this, consider only one radius range containing one cell of type A with or without one cell of type C and that we are trying to evaluate the probability λ(x,B) that a different cell in that radius is of type B. Let δ_AB_ = 0.1 (signifying repulsion; note δ_AB _= δ_BA_) and δ_BC_ = 20 (signifying attraction). Then λ(x,B) ~ γ_AB_*γ_BC_ and, since A is inside the radius (in both cases), γ_AB_ = δ_AB_ = 0.1. If C is not present (not inside the radius), γ_AC_ = 1 giving λ(x,B) ~ (0.1)(1) = 0.1. If C is present (inside the radius), γ_AC_ = δ_AC_ = 20 and λ(x,B) ~ (0.1)(20) = 2. Thus A attracts B if C is present, but repels B if C is not present. Changing δ_AB_ to 2 and δ_BC_ to 0.05 reverses this “multipartner” relationship.

Second, the model can capture nonlinear relationships arising from differences in intensity in different ranges. For single range models, probabilities can only be linear in intensity. However, in our multirange model, changes in relative intensity in different ranges can cause nonlinear effects. Referring to equation 5, if the δ_AB_ for two ranges differ significantly, the net contribution to λ(x,B) will depend on the densities (number of cells) of A in each range. For example, if n_A1_ is the number of A in range 1, λ(x,B) ~ δ_AB1_^na1^ * δ_AB2_^(n-na1)^. If one of the δ are greater than 1 and the other is less than 1, the effect of A on the probability of B can go up or down depending on the distribution of A between ranges. Additionally, effects can be seen from variation in density of a third type in different ranges (similar to the multipartner effect above).

### Training the point process model

The standard method of fitting point process models to existing data utilizes maximum likelihood estimation (MLE). However, it’s difficult to calculate or approximate the normalizing constant α in the probability density function *f* [57]. As an alternative we calculated the log pseudolikelihood:


$$\text{log}\;PL\left(\theta ,p\right)=\sum {\mathrm{\backslash nolimits}}_{i=1}^{n}\text{log}\;\lambda_{\theta }\left(\left(x_{i},\;c_{i}\right)|p\right)-\sum {\mathrm{\backslash nolimits}}_{c}\int_{W}\lambda_{\theta }\left((u\text{,}\;c)|p\right)du$$ (6)

where θ = (β, δ) is a set of coefficients we need to estimate where $\beta =\left(\beta_{c_{i}}\right),\;c_{i}\in C$ is the first-order term or intensity of each cell type and $\delta ={(\delta }_{c_{i},c_{j}}boldsymbol,\;c_{i},c_{j}\in C$ is the set of interaction coefficients between each pair of cell types, *W* is the image window, and the integration is on all possible points u over all possible cell types c within this window given the point pattern ***p***.

The difficulty of estimating maximum pseudolikelihood is the computational infeasibility of integrating over every location within the image window. Therefore, we applied the Berman-Turner quadrature scheme [57,58] to approximate the background distribution of the conditional intensity function. Each image was evenly split into subregions (tiles) using Dirichlet tesselation. At the center of each tile and four corners of the image, dummy cells for each of the cell types were created. At the location of each real cell, dummy cells for all cell types except the real cell type were also created. This way the integration was converted to a sum weighted by the intensity of cells. The intensity of a cell was calculated by the ratio of the number of cells in its tile to the size of the tile. In other words, cells in the same tile have the same intensity. The approximate log pseudolikelihood is then:


$$\text{log}\;PL\left(\theta ,p\right)\approx \sum {\mathrm{\backslash nolimits}}_{i=1}^{n}\text{log}\;\lambda_{\theta }\left(\left(x_{i},\;c_{i}\right)|p\right)-\sum {\mathrm{\backslash nolimits}}_{c}\sum {\mathrm{\backslash nolimits}}_{j=1}^{n^{\prime }}\;w_{j}\lambda_{\theta }\left({{(x}^{\prime }}_{j},{c^{\prime }}_{j})\;|p^{\prime }\right)$$ (7)

where $p^{\prime }$ is the new point pattern generated by the quadrature scheme that includes both real and dummy cells, $n^{\prime }$ is the total number of real and dummy cells, and weight $\text{\textbackslash{},}w_{j}$ is calculated by the area of a quadrature grid over the number of cells in the grid.

We then performed maximum pseudolikelihood estimation by generalized linear model (GLM). The first step was to construct a feature matrix for GLM’s regression (see S2 Table). For each point, we counted the number of neighboring cells within a specified distance (multirange Strauss radius). Fitting is done with the R generalized linear model fitting module “glm” using the quasi-likelihood family with link = “log” and variance = “mu”. The label to predict was the local intensity $y_{i}=I_{i}/w_{i}$, where $I_{i}$ is an indicator function that equals 1 if current cell is real and 0 if it is a dummy [59,60]. The whole training process was done by modifying the R package spatstat [61]. We created a new function for our multirange, multitype model.

Computational complexity of model learning by CytoSpatio is O(number of cells*number of radii*number of types^2^). For 50,000 cells, a typical run time on a single cpu is approximately 25 minutes for 5 types and 5 radii, thus estimated run times are (number of cells/50000)(number of radii/5)*(number of types)^2^.

### Error metric of point process model

Pseudolikelihood can appropriately be used to compare different models trained on the same point pattern. However, pseudolikelihoods for models trained on different patterns are not comparable since those patterns may contain different numbers of cells.

To obtain an error metric that is independent of the training data size, we rewrite the pseudolikelihood as:


$$log\;PL\left(\theta ;p\right)=-\frac{D}{2}+g$$ (8)

where *g* is a constant and therefore irrelevant in pseudolikelihood comparison. *D* is the deviance that can be written as:


$$D=2\left(\log PL_{s}\left(y\right)-\log PL\left(\hat{\theta }\right)\right)=2\sum_{i=1}^{n^{D}}w_{i\;}\left(y_{i}\log\left(y_{i}/\mu_{i}\right)-\left(y_{i}-\mu_{i}\right)\right)$$

μ=exp(η) (9)

$$\eta ={\hat{\theta }}^{T}X$$

where $log\;PL_{s}\left(y\right)$ is the log pseudolikelihood of a “saturated” model that has one parameter for each cell to achieve a perfect fit for the data, $log\;PL\left(\hat{\theta }\right)$ is the log pseudolikelihood of the model under estimation,$n^{\text{D}}$ is the number of cells being included (either equal to *n* to calculate just for real cells, $n^{\prime }-n$ to calculate for just dummy cells, or $n^{\prime }$ to calculate for both), $w_{i\;}$ is weight for cell *i* (definition same as in the equation of log pseudolikelihood), $y_{i}=\frac{I_{i}}{w_{i}}$ is the true label (as above, the local intensity for real cells and 0 otherwise) and $\mu_{i}$ is the predicted label for cell *i* in GLM. ***X*** is the input feature matrix, $\hat{\theta }$ is a vector that contains all base intensity coefficients and interaction coefficients need to be estimated. We assumed the model belongs to the exponential family. We therefore applied an exponential as the link function of GLM between the linear product η and predicted label μ.

To account for the influence of data size, we normalized deviance *D* by dividing it by the cell number *n*, yielding the average deviance per cell as our error metric. We interpreted this metric as the average difference between the observed local intensity for each cell and its predicted intensity from a trained model. This metric is particularly sensitive to the value of η. An increase in η would exponentially elevate μ, leading to a significantly higher average deviance per cell, as seen in Table 1.

### Leave-one-out cross-validation

To prevent overfitting when comparing point process models trained on different tissues, we conducted a leave-one-out cross-validation for each tissue. In this process, we sequentially excluded one image from the current tissue’s training set, fit the model to the remaining images, and predicted the average deviance per cell for the left-out image. As a result, the number of models for each tissue equaled the number of images. We used them as an ensemble representation of their respective tissues only for the analysis of cell type prediction accuracy in the following section.

### Assessing cell type prediction accuracy

We utilized the Receiver Operating Characteristic (ROC) curve, which is derived from the false positive rate and the true positive rate, to measure the accuracy of cell type prediction. Given that we have five cell types, we need a multi-class ROC; for this, a prediction for one cell type was considered true only if it matched the corresponding cell type and false otherwise.

To calculate overall prediction accuracy, we employed several techniques. First, we calculated the Micro AUC, which considered each cell (independent of its actual type) and counted whether it was correctly predicted. However, a potential issue with Micro AUC arises when class imbalance exists. If a majority of the predictions are biased towards the majority class, Micro AUC could be misleadingly high. This is because the true positive rate and false positive rate in Micro AUC are derived from aggregating predictions across all classes. Consequently, strong performance on the majority class can significantly overshadow any poor performance on the minority classes.

We also computed the Macro AUC to evaluate each cell type independently. This method computes the AUC separately for each class and then averages them, giving equal weight to each class. However, Macro AUC can also be less representative of the model’s overall performance when the class frequencies are different. If a model performs well on a minority class but poorly on a majority class, the Macro AUC might still appear reasonably high despite the model’s overall lower performance on most instances.

We therefore adopted the Weighted Macro AUC (wmAUC) to address this class imbalance issue. Like the Macro AUC, this approach evaluates each cell type independently, but it counters class imbalances by weighting the AUC of each cell type according to its fraction within the total number of cells. Thus, if certain cell types are more common in the dataset, they are assigned more importance in the overall score calculation. Given its effective solution to class imbalance, we chose to use this metric to evaluate the prediction accuracy of cell types.

### Generating synthetic tissue images

To begin generating synthetic images, we generated cell centers using a Poisson distribution that maintained the same total cell density as the original image. From this, synthetic cell shapes were created using a Voronoi diagram truncated at 20 pixels (approximately 7.5 micron radius). Synthetic images were then created from this arrangement by two methods using our models and two baseline methods for comparison. For method 1, cell types were randomly assigned cell types based on the density/frequency of each cell type in the real images. Following this, we randomly and iteratively selected a cell and reassigned its type according to the cell type counts for that location and the likelihoods derived from the model (the model in this case was for one original image of each tissue). This process was continued until the number of sampled cells reached a specified percentage of the total cell count in the image. For method 2, cells were randomly chosen one cell at a time, and the type of that cell and its neighbors were assigned according to the model. A score between 0 and 1 reflecting the agreement between the assigned cell types and the neighbor frequencies predicted by the model was calculated, and the sampling process continued until a threshold of 0.9 was passed for 1000 consecutive iterations. For comparison, images were also generated from the initial synthetic cell arrangement by assigning cell types randomly either according to their frequencies (like the initial step of Method 1) or according to equal frequencies.

To provide an independent measure of how well the synthetic cell arrangements agreed with those of real images, each synthetic or real image was represented by a matrix in which each element of the matrix contains the count of cells of a given type (row) that are within 100 pixels of a cell of another type (column). The Euclidean distances between the matrices was calculated for all pairs of synthetic images from a given method with all pairs of real images (for this purpose, regions of the same size were randomly chosen from real images).

### Data and code availability

- CytoSpatio software is available at https://github.com/murphygroup/CytoSpatio.
- All data used for this work are available as a reproducible research archive (https://github.com/murphygroup/ChenMurphyCytoSpatioRRA).

## Supporting information

S1 Fig Self-interactions of five cell types across five different tissues. Each node represents the self-interactions of one cell type. The self-interaction range, which increases from bottom to top, is divided into five arcs. The size of each node corresponds to the total strength of self-interaction for that cell type. The strength of the self-interaction relationship is depicted by the thickness of the arc. The nature of the interaction is indicated by the color of the arc, with blue as attraction and red as repulsion.
(TIFF)

S2 Fig Cell type interaction graph for five cell types across five different tissues filtered to remove interactions whose 95% confidence interval includes 0. Each graph is shows raw interaction strength as shown in Fig 5 but with edges that are not significantly different from 0 removed.
(TIFF)

S3 Fig Heatmaps of pairwise interaction scores for the IMC dataset. From left to right and top to bottom, the panels correspond to increasing ranges from 37.7 to 187.5 microns. Each panel shows the median interaction score between a given pair of cell types across all 59 images (blue is negative/repulsion). As expected, the magnitude of interaction generally diminishes with increasing distance, reflecting a decay in spatial association at broader ranges.
(TIFF)

S4 Fig Evaluation of synthetic tissue image simulation. The weighted macro AUCROC of synthetic images generated using random Poisson cell locations are shown after various amounts of resampling for five tissue types. Each curve plotted corresponds to a synthetic image generated by a model that was trained on an original tissue image. The ‘resampling percent’ refers to the percentage of the total cell count that were randomly sampled and reassigned according to the model.
(TIFF)

S5 Fig Quantitative comparison of synthetic images with real images. The panel for each tissue shows a box-and-whisker plot of the Euclidean distances of each of 10 synthetic images generated by a particular method with each of 10 real images (and 10 real images with each other). Each synthetic or real image was represented by a matrix in which each cell contains the count of cells of a given type (row) that are within 100 pixels of a cell of another type (column). The plots in each panel from left to right show Methods 1 and 2 (described in the Materials and methods), assigning cell types randomly according to their frequencies, assigning cell types randomly with equal frequencies, and real images.
(TIFF)

S6 Fig Interaction graphs for three different distance ranges. From left to right are models with ranges from 50 to 500 by 50, 100–500 by 100, and 200–400 by 200. Note that the types of interactions (repulsion edges shown in red, attraction edges shown in blue) remain relatively consistent. The relative thickness of edges increases with interval since it reflects the area of the range.
(TIFF)

S7 Fig Gaussian Kernel similarity between cluster centroids. Shown are (A) each pair of clusters resulting from KMeans and (B) between each KMeans cluster and each cell type from Cellar. A lighter color indicates higher similarity. KMeans clusters with no cells were excluded from the similarity calculation.
(TIFF)

S8 Fig Defining cell types by comparing cell intensities with Cellar annotations. (A) Determination of the optimal number of clusters in KMeans for cell type definition. The number of clusters was gradually increased until the majority of four Cellar-annotated cell types (“other cells” excluded) showed a consistent cell count. CD4-positive T cells proved the most challenging to identify. We chose 39 as the best cluster number since it presented cell counts most aligned with Cellar annotations, as indicated by the final point on the x-axis. The colors of cell types are consistent with S1 Fig. (B) Comparison of our cell type identification and Cellar annotation. Our approach yielded cell counts similar to Cellar annotations with slightly higher numbers for each of cell types. This variation is due to our identification using only 5 shared channels across the five tissue types for cell type classification, in contrast to the 19 channels utilized in Cellar.
(TIFF)

S1 Table Summary of tissue images by type and source. (PDF)

S2 Table Contents of the concatenated feature matrix assembled for model fitting. Fields in the data structure for each cell are created by combining the results from the quadrature schemes for different ranges. The results for a given set of images are then concatenated using pattern_ID to distinguish the source image. Model fitting is done using the R generalized linear fitting module “glm” with the formula mpl.Y ~ marks + Interactionmarks, and each cell is weighted by mpl.W * caseweight.
(PDF)

S3 Table Comparison of fitted models for different range specifications. Models were run with ranges 100–500 by 100, 200–400 by 200, and 50–500 by 50. The resulting coefficients are shown for specific ranges.
(PDF)

S4 Table Examples of “multipartner” interactions observed in tissue models. All examples are from range 100. These examples assume roughly equal number of the three partners being present. The effects may be modulated by differences in frequency of the types.
(PDF)
