# Supplementary figures and images for: CytoSpatio: Learning cell type spatial relationships using multirange, multitype point process models
Source: PLoS Comput Biol. 2025 Aug 21;21(8):e1013409. doi: 10.1371/journal.pcbi.1013409 (PMC12396756; doi:10.1371/journal.pcbi.1013409)

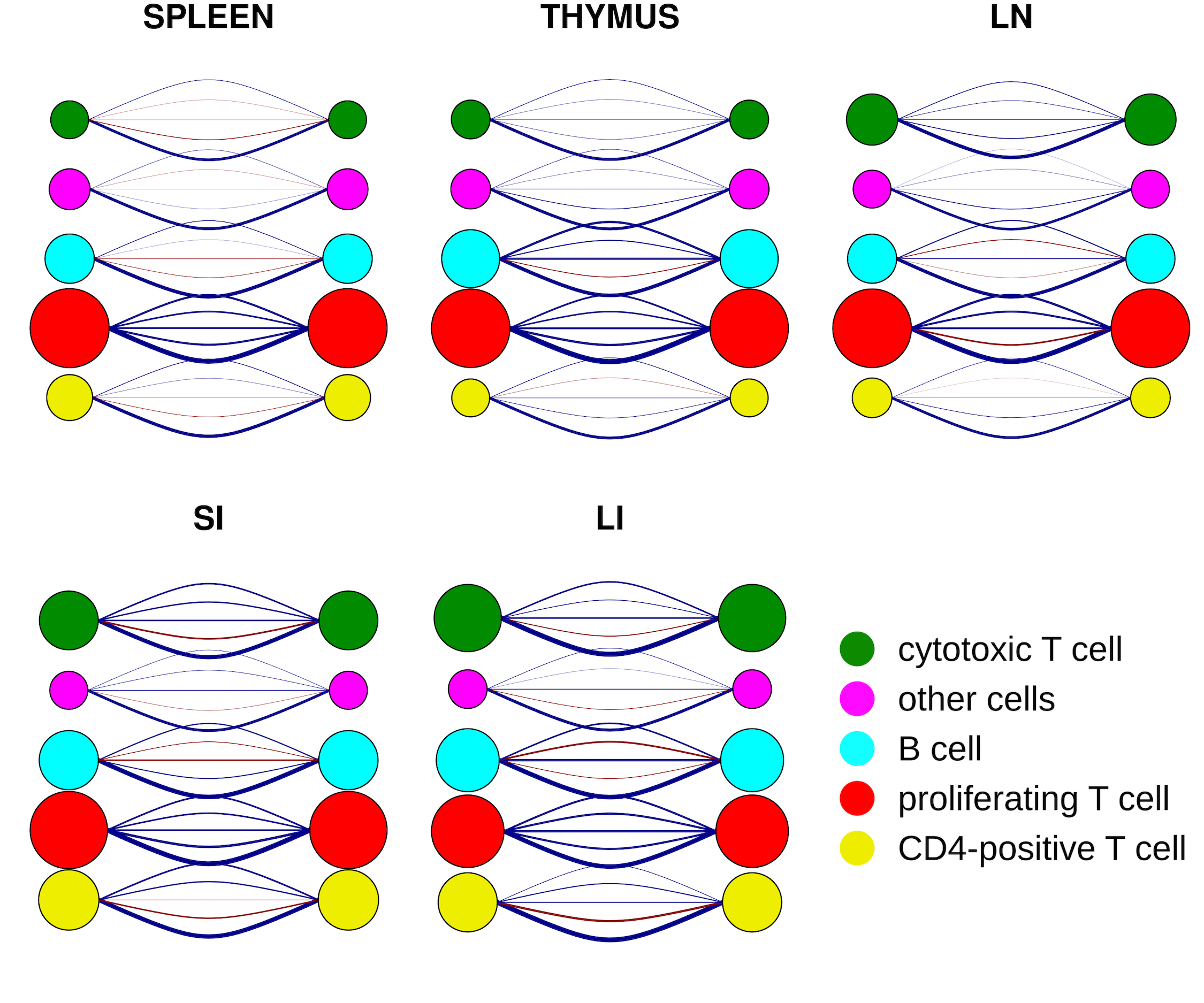

Supplement: S1 Fig — Each node represents the self-interactions of one cell type. The self-interaction range, which increases from bottom to top, is divided into five arcs. The size of each node corresponds to the total strength of self-interaction for that cell type. The strength of the self-interaction relationship is depicted by the thickness of the arc. The nature of the interaction is indicated by the color of the arc, with blue as attraction and red as repulsion. (TIFF) [file pcbi.1013409.s001.tiff]

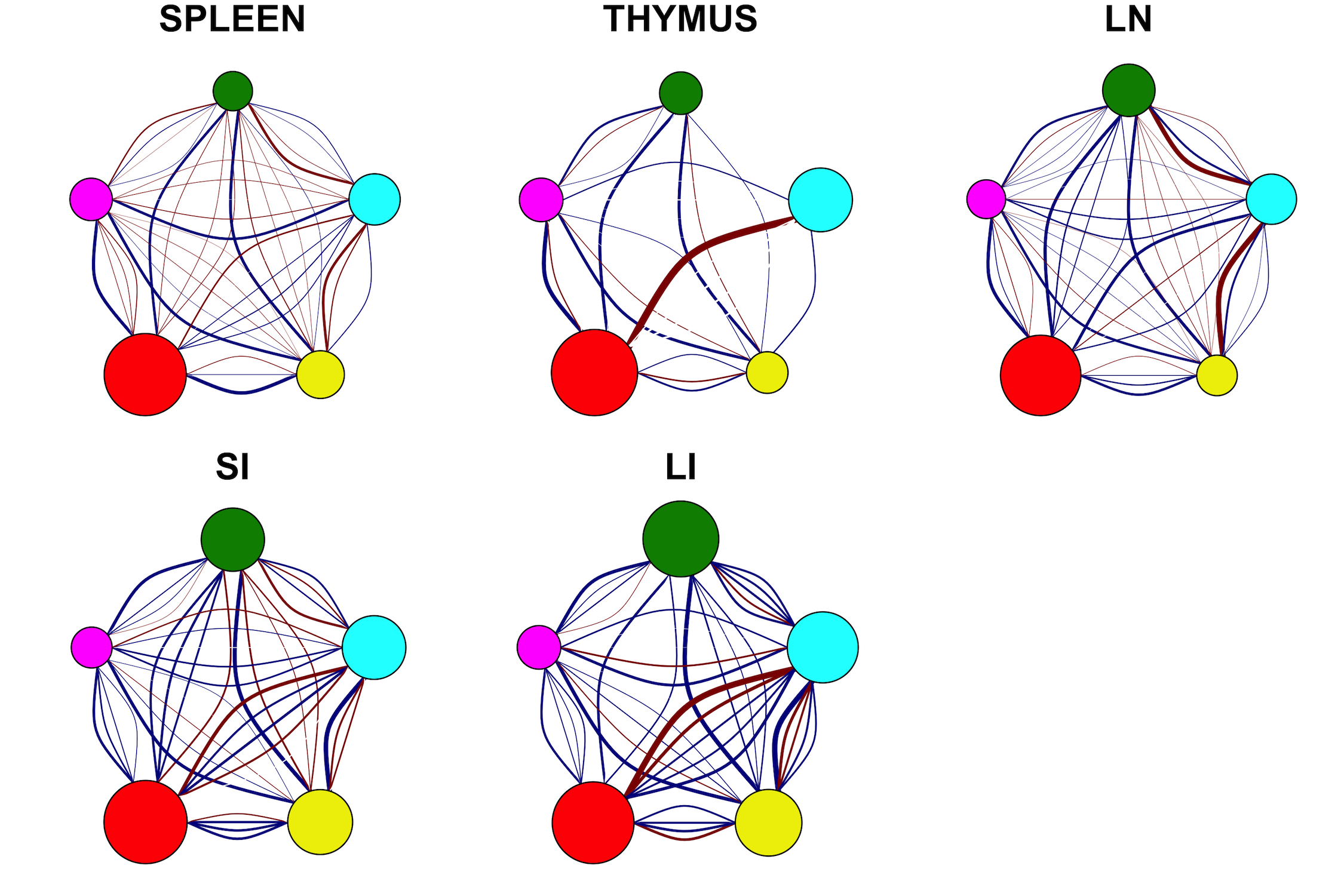

Supplement: S2 Fig — Each graph is shows raw interaction strength as shown in Fig 5 but with edges that are not significantly different from 0 removed. (TIFF) [file pcbi.1013409.s002.tiff]

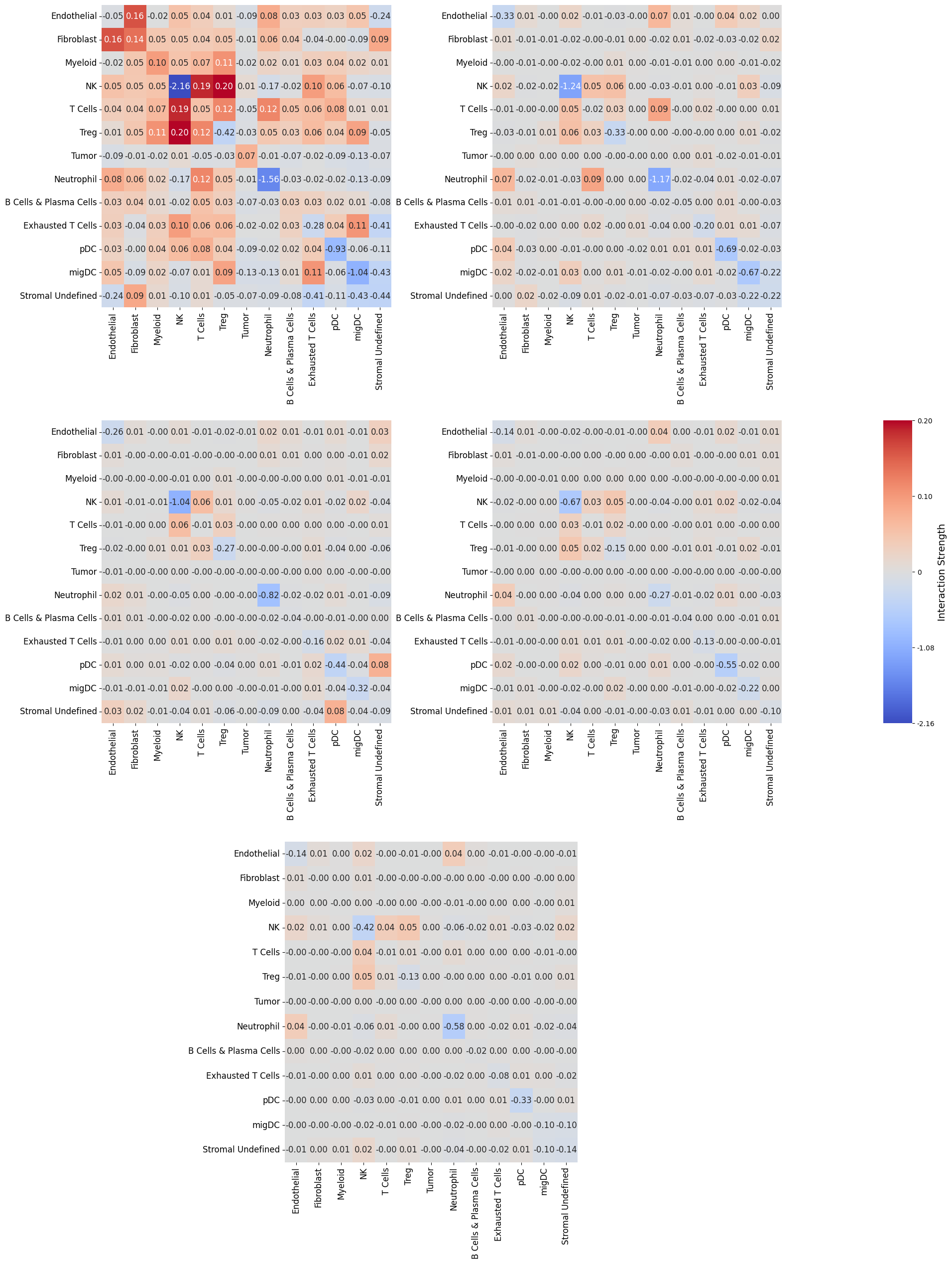

Supplement: S3 Fig — From left to right and top to bottom, the panels correspond to increasing ranges from 37.7 to 187.5 microns. Each panel shows the median interaction score between a given pair of cell types across all 59 images (blue is negative/repulsion). As expected, the magnitude of interaction generally diminishes with increasing distance, reflecting a decay in spatial association at broader ranges. (TIFF) [file pcbi.1013409.s003.tiff]

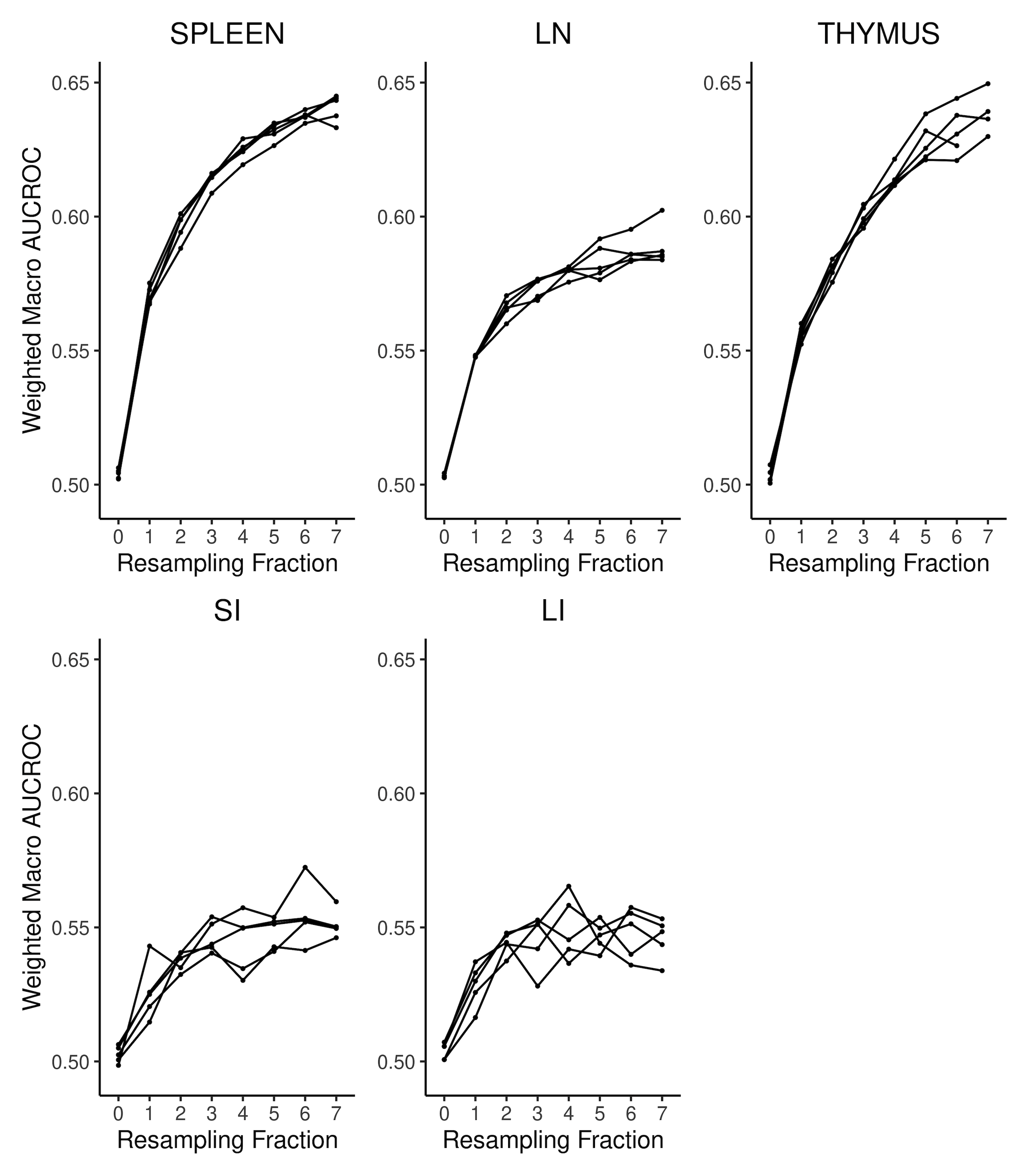

Supplement: S4 Fig — The weighted macro AUCROC of synthetic images generated using random Poisson cell locations are shown after various amounts of resampling for five tissue types. Each curve plotted corresponds to a synthetic image generated by a model that was trained on an original tissue image. The ‘resampling percent’ refers to the percentage of the total cell count that were randomly sampled and reassigned according to the model. (TIFF) [file pcbi.1013409.s004.tiff]

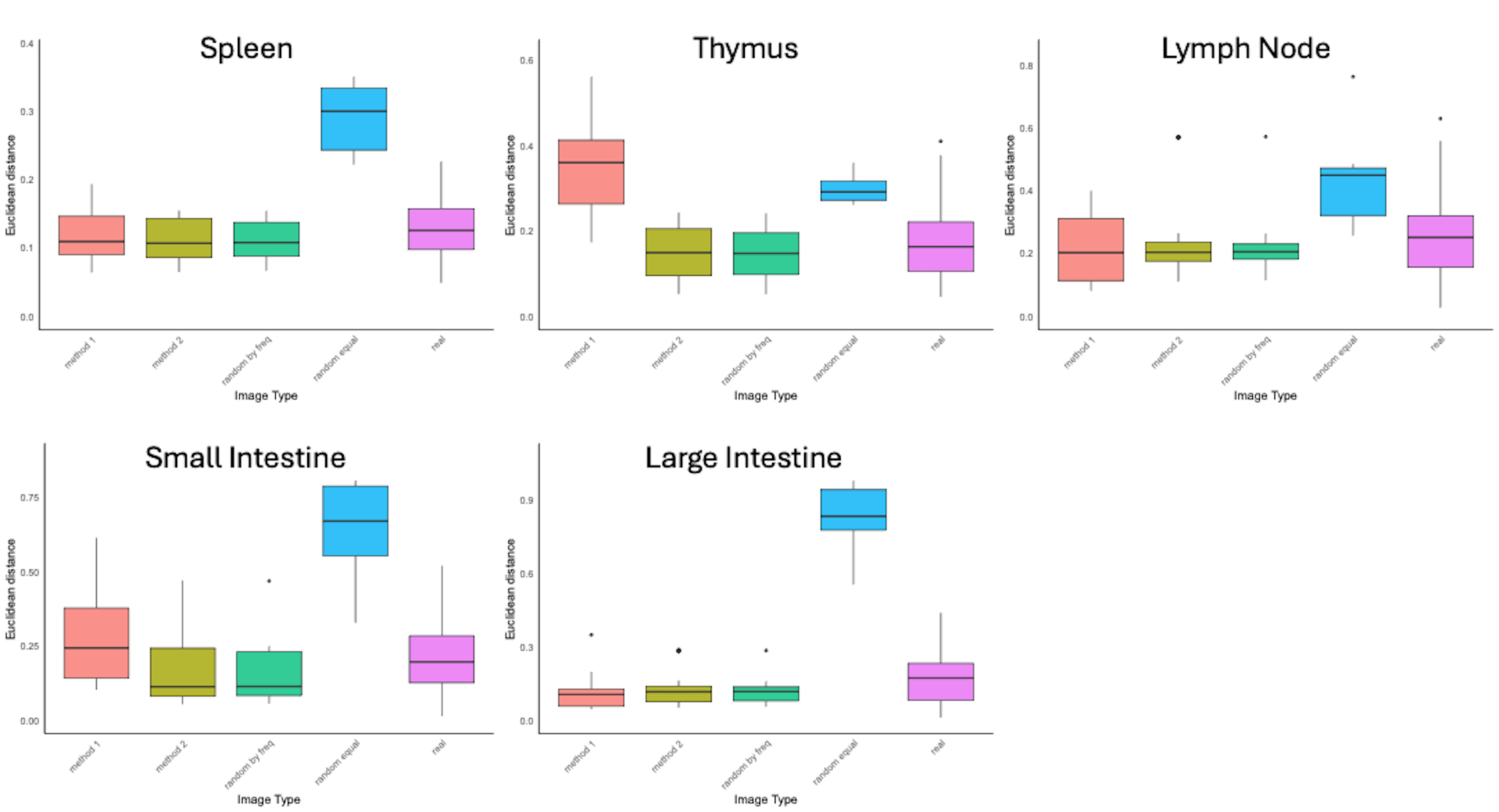

Supplement: S5 Fig — The panel for each tissue shows a box-and-whisker plot of the Euclidean distances of each of 10 synthetic images generated by a particular method with each of 10 real images (and 10 real images with each other). Each synthetic or real image was represented by a matrix in which each cell contains the count of cells of a given type (row) that are within 100 pixels of a cell of another type (column). The plots in each panel from left to right show Methods 1 and 2 (described in the Materials and methods), assigning cell types randomly according to their frequencies, assigning cell types randomly with equal frequencies, and real images. (TIFF) [file pcbi.1013409.s005.tiff]

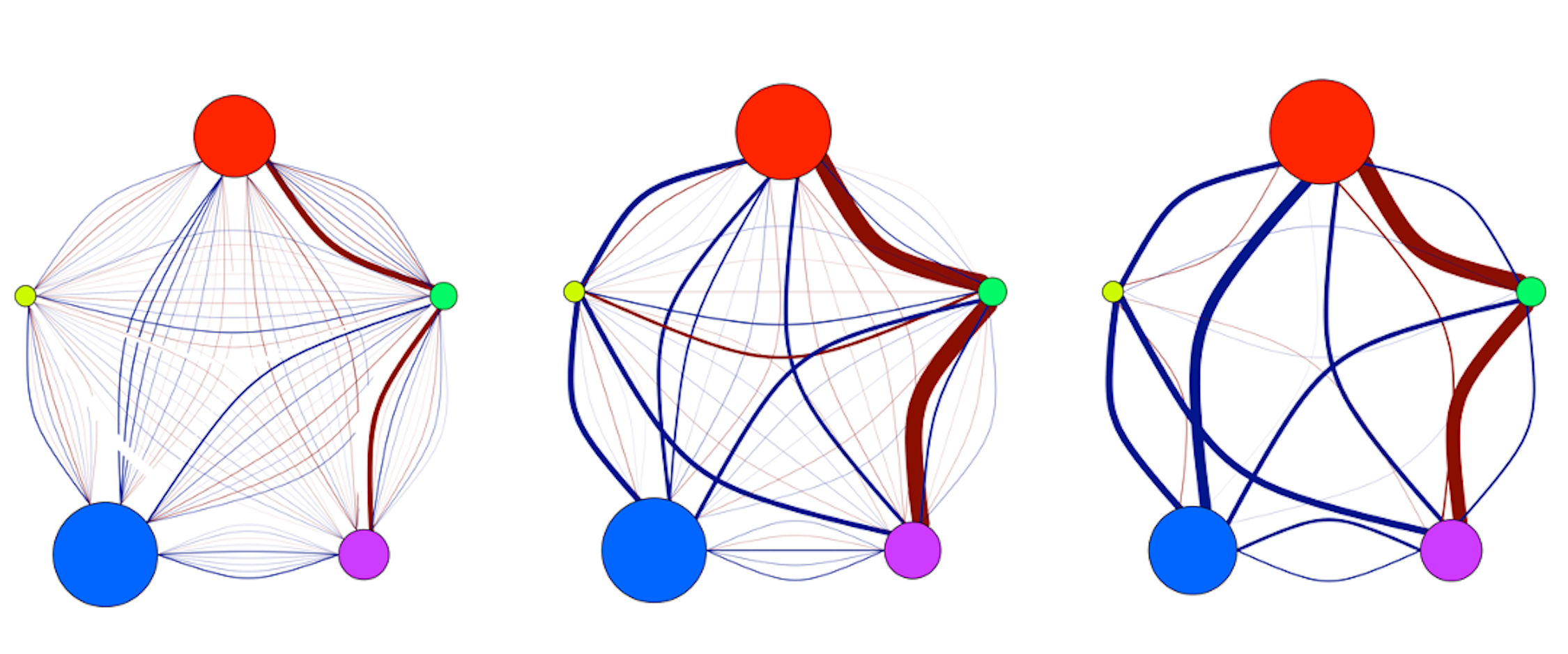

Supplement: S6 Fig — From left to right are models with ranges from 50 to 500 by 50, 100–500 by 100, and 200–400 by 200. Note that the types of interactions (repulsion edges shown in red, attraction edges shown in blue) remain relatively consistent. The relative thickness of edges increases with interval since it reflects the area of the range. (TIFF) [file pcbi.1013409.s006.tiff]

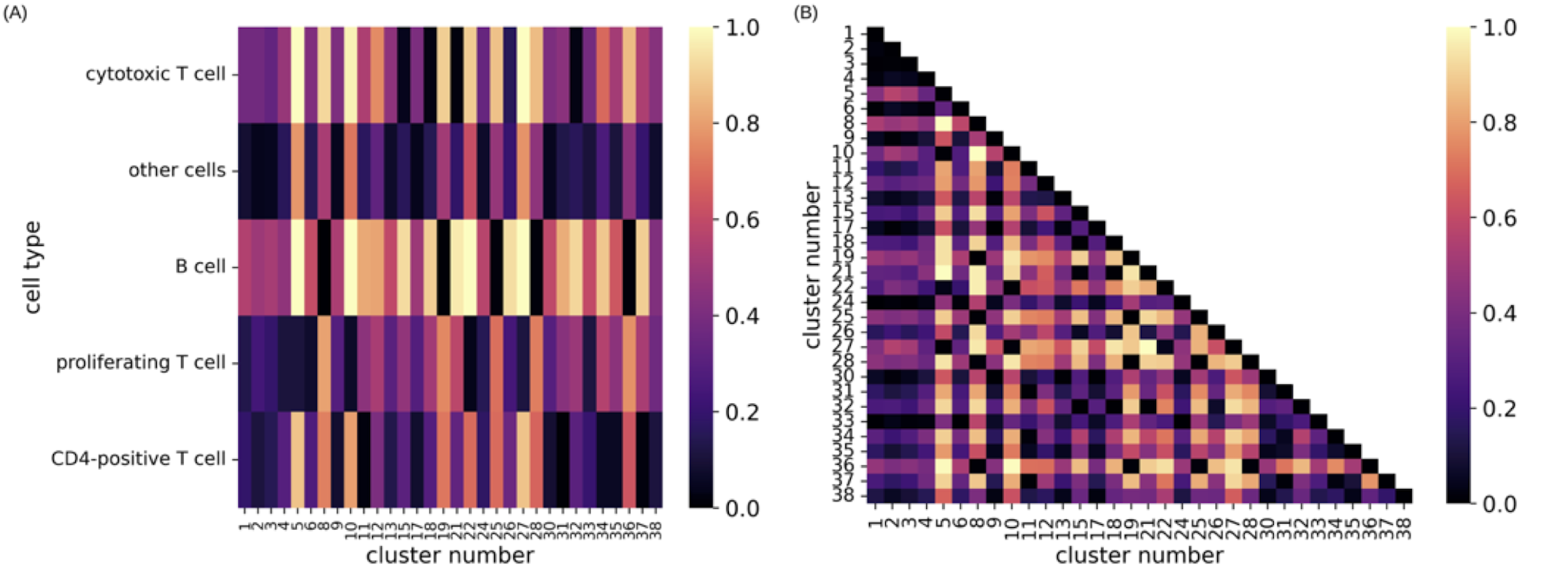

Supplement: S7 Fig — Shown are (A) each pair of clusters resulting from KMeans and (B) between each KMeans cluster and each cell type from Cellar. A lighter color indicates higher similarity. KMeans clusters with no cells were excluded from the similarity calculation. (TIFF) [file pcbi.1013409.s007.tiff]

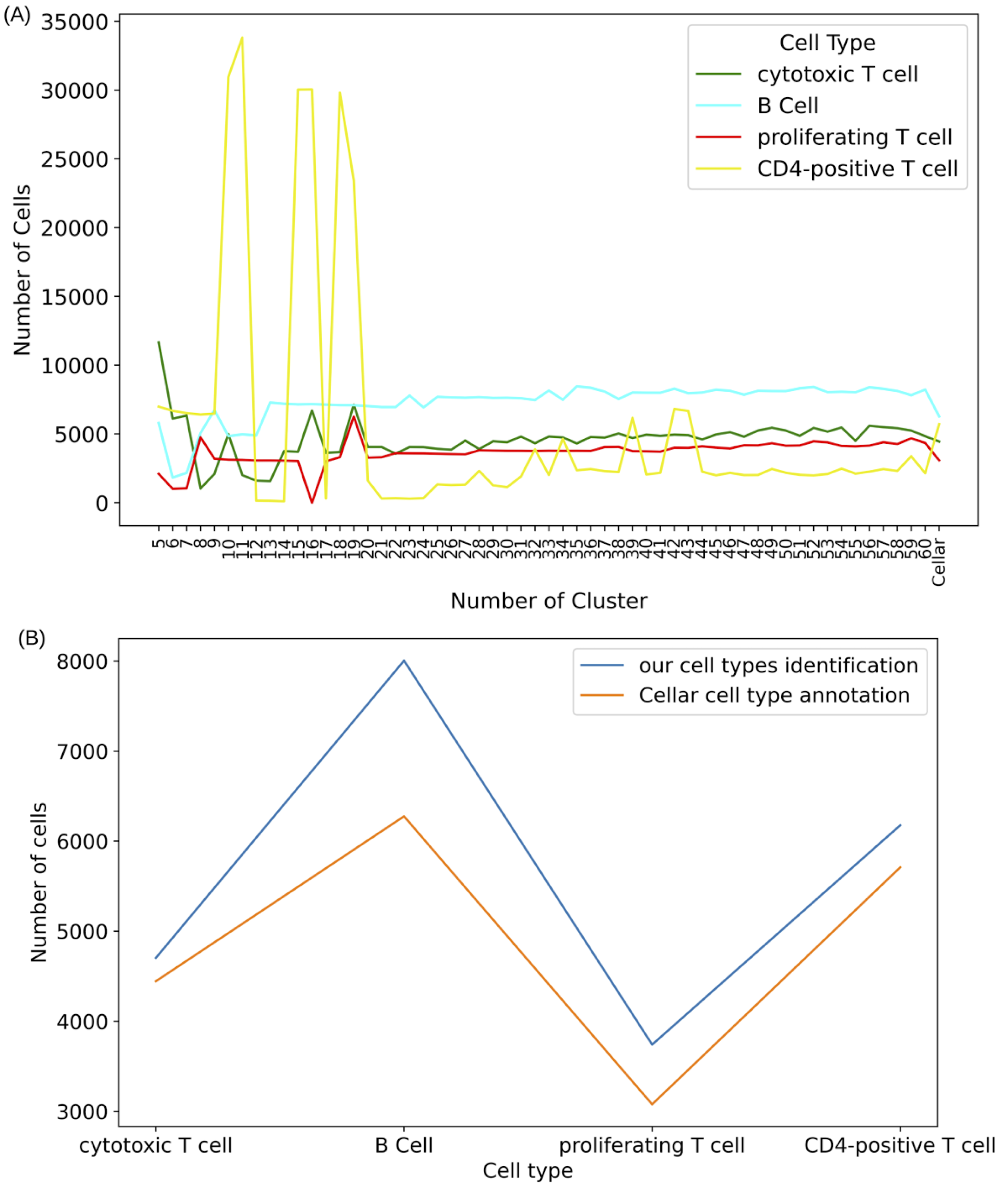

Supplement: S8 Fig — (A) Determination of the optimal number of clusters in KMeans for cell type definition. The number of clusters was gradually increased until the majority of four Cellar-annotated cell types (“other cells” excluded) showed a consistent cell count. CD4-positive T cells proved the most challenging to identify. We chose 39 as the best cluster number since it presented cell counts most aligned with Cellar annotations, as indicated by the final point on the x-axis. The colors of cell types are consistent with S1 Fig. (B) Comparison of our cell type identification and Cellar annotation. Our approach yielded cell counts similar to Cellar annotations with slightly higher numbers for each of cell types. This variation is due to our identification using only 5 shared channels across the five tissue types for cell type classification, in contrast to the 19 channels utilized in Cellar. (TIFF) [file pcbi.1013409.s008.tiff]
