# Supplementary material for: CytoSpatio: Learning cell type spatial relationships using multirange, multitype point process models
Source: PLoS Comput Biol. 2025 Aug 21;21(8):e1013409. doi: 10.1371/journal.pcbi.1013409 (PMC12396756; doi:10.1371/journal.pcbi.1013409)
Supplement: S1 Table — (PDF) [file pcbi.1013409.s009.pdf]

S1 Table. Summary of tissue images by type and source

Tissue type	Number of images	Tissue Mapping Center
Large Intestine (LI)	26	Stanford
Small Intestine (SI)	22	
Lymph Node (LN)	26	University of Florida
Spleen	24	
Thymus	12	
