# Supplementary material for: CytoSpatio: Learning cell type spatial relationships using multirange, multitype point process models
Source: PLoS Comput Biol. 2025 Aug 21;21(8):e1013409. doi: 10.1371/journal.pcbi.1013409 (PMC12396756; doi:10.1371/journal.pcbi.1013409)
Supplement: S2 Table — Fields in the data structure for each cell are created by combining the results from the quadrature schemes for different ranges. The results for a given set of images are then concatenated using pattern_ID to distinguish the source image. Model fitting is done using the R generalized linear fitting module “glm” with the formula mpl.Y ~ marks + Interactionmarks, and each cell is weighted by mpl.W * caseweight. (PDF) [file pcbi.1013409.s010.pdf]

**S2 Table.** Contents of the concatenated feature matrix assembled for model fitting.

| Field | Description | Used in fitting |
|---|---|---|
| CellID | Unique number for each cell | No |
| .mpl.W | The weight to apply to this cell | Yes |
| .mpl.Y | 1 / .mpl.W for real cells and 0 for dummy cells | Yes |
| x | X coordinate of cell in original image | No |
| y | Y coordinate of cell in original image | No |
| marks | The type of this cell | Yes |
| .mpl.SUBSET | Indicator of which cells should be included in fitting, always TRUE (input argument to | Yes |
| InteractionmarkX***ii***xX***jj***x***rr*** | Count of interactions between type ***ii*** and type ***jj*** in range ***rr*** (e.g., InteractionmarkX0xX1x100 for interaction between type 0 and type 1 in range 100) | Yes |
| pattern_ID | Indicator of the image source of each cell. If one image, always 1; if N images, cells from the same image share the same pattern ID, from 1 to N. | No |
| caseweight | Always 1 | Yes |

Fields in the data structure for each cell are created by combining the results from the quadrature schemes for different ranges. The results for a given set of images are then concatenated using pattern_ID to distinguish the source image. Model fitting is done using the R generalized linear fitting module “glm” with the formula mpl.Y ~ marks + Interactionmarks, and each cell is weighted by mpl.W * caseweight.
