# Supplementary material for: CytoSpatio: Learning cell type spatial relationships using multirange, multitype point process models
Source: PLoS Comput Biol. 2025 Aug 21;21(8):e1013409. doi: 10.1371/journal.pcbi.1013409 (PMC12396756; doi:10.1371/journal.pcbi.1013409)
Supplement: S3 Table — Models were run with ranges 100–500 by 100, 200–400 by 200, and 50–500 by 50. The resulting coefficients are shown for specific ranges. (PDF) [file pcbi.1013409.s011.pdf]

S3 Table. Comparison of fitted models for different range specifications.

	100:100:500 range 0:100	200:200:400 range 0:200	50:50:500 range 0:50	50:50:500 range 50:100
**a) betas (base intensities)**				
0	-10.325	-10.272	-10.353	
1	1.274	1.255	1.278	
2	0.511	0.502	0.540	
3	-0.416	-0.448	-0.394	
4	0.076	0.046	0.090	
average of ratio to 0:100		0.928	1.039	
sd of ratio		0.186	0.091	
correlation w/ 0:100		1.000	1.000	
**b) interaction coefficients**				
0x0	0.099	0.039	0.263	0.028
0x1	0.023	0.005	0.065	0.004
1x1	0.052	0.018	0.140	0.015
0x2	-0.070	-0.015	-0.122	-0.047
1x2	-0.012	0.000	-0.036	-0.002
2x2	0.058	0.021	0.156	0.016
0x3	0.015	0.010	0.032	0.009
1x3	0.023	0.006	0.058	0.008
2x3	0.016	0.004	0.015	0.014
3x3	0.129	0.037	0.385	-0.004
0x4	0.015	0.004	0.054	-0.001
1x4	0.020	0.006	0.071	0.000
2x4	-0.068	-0.012	-0.128	-0.037
3x4	0.004	0.002	-0.011	0.008
4x4	0.070	0.025	0.193	0.023
average abs value	0.045	0.014	0.115	0.014
average of ratio to 0:100		0.300	2.205	1.321
correlation w/ 0:100		0.976	0.984	0.994

Models were run with ranges 100 to 500 by 100, 200 to 400 by 200, and 50 to 500 by 50. The resulting coefficients are shown for specific ranges.
