# Supplementary material for: CytoSpatio: Learning cell type spatial relationships using multirange, multitype point process models
Source: PLoS Comput Biol. 2025 Aug 21;21(8):e1013409. doi: 10.1371/journal.pcbi.1013409 (PMC12396756; doi:10.1371/journal.pcbi.1013409)
Supplement: S4 Table — All examples are from range 100. These examples assume roughly equal number of the three partners being present. The effects may be modulated by differences in frequency of the types. (PDF) [file pcbi.1013409.s012.pdf]

S4 Table. Examples of “multipartner” interactions observed in tissue models.

LI
CD4-positive T cell is attracted to lymphocyte of B lineage with coefficient 0.0845791623566985 unless proliferating T cell is present (with coefficient -0.0174001486344555) (net=0.067179013722243).
lymphocyte of B lineage is attracted to cytotoxic T cell with coefficient 0.0127683877761322 unless proliferating T cell is present (with coefficient -0.110757862131299) (net=-0.0979894743551668).
SI
lymphocyte of B lineage is attracted to CD4-positive T cell with coefficient 0.0574357283057224 unless cytotoxic T cell is present (with coefficient -0.0140905503751541) (net=0.04334517793056829 5).
LN
CD4-positive T cell is attracted to proliferating T cell with coefficient 0.013807132294015 unless lymphocyte of B lineage is present (with coefficient -0.0870918322315641) (net=-0.0732846999375491).
THYMUS
proliferating T cell is attracted to cytotoxic T cell with coefficient 0.0231546674288584 unless lymphocyte of B lineage is present (with coefficient -0.167735886526695) (net=-0.14458121909783658).
proliferating T cell is attracted to other cells with coefficient 0.044845867036508 unless lymphocyte of B lineage is present (with coefficient -0.167735886526695) (net=-0.12289001949018699).
SPLEEN
proliferating T cell is attracted to CD4-positive T cell with coefficient 0.0314820544838602 unless lymphocyte of B lineage is present (with coefficient -0.0048437638969577) (net=0.026638290586902497).

All examples are from range 100. These examples would occur if roughly equal numbers of the three partners were present, and may be modulated by differences in frequency of the types.
